# A Novel Nitrogen and Carbon Metabolism Regulatory Cascade Is Implicated in Entomopathogenicity of the Fungus Metarhizium robertsii

**DOI:** 10.1128/mSystems.00499-21

**Published:** 2021-06-22

**Authors:** Yamin Meng, Xing Zhang, Dan Tang, Xiaoxuan Chen, Dan Zhang, Jun Chen, Weiguo Fang

**Affiliations:** a MOE Key Laboratory of Biosystems Homeostasis & Protection, Institute of Microbiology, College of Life Science, Zhejiang University, Hangzhou, China; b Laboratory of Systematic and Evolutionary Botany and Biodiversity, College of Life Sciences, Zhejiang University, Hangzhou, China; UiT—The Arctic University of Norway; South China Agricultural University

**Keywords:** entomopathogenic fungi, *Metarhizium*

## Abstract

The entomopathogenic fungus Metarhizium robertsii can switch among parasitic, saprophytic, and symbiotic lifestyles in response to changing nutritional conditions, which is attributed to its extremely versatile metabolism. Here, we found that the Fus3–mitogen-activated protein kinase (MAPK) and the transcription factor regulator of nutrient selection 1 (RNS1) constitute a novel fungal cascade that regulates the degradation of insect cuticular lipids, proteins, and chitin to obtain nutrients for hyphal growth and enter the insect hemocoel for subsequent colonization. On the insect cuticle, Fus3-MAPK physically contacts and phosphorylates RNS1, which facilitates the entry of RNS1 into nuclei. The phosphorylated RNS1 binds to the DNA motif *BM2* (ACCAGAC) in its own promoter to self-induce expression, which then activates the expression of genes for degrading cuticular proteins, chitin, and lipids. We further found that the Fus3-MAPK/RNS1 cascade also activates genes for utilizing complex and less-favored nitrogen and carbon sources (casein, colloid chitin, and hydrocarbons) that were not derived from insects, which is repressed by favored organic carbon and nitrogen nutrients, including glucose and glutamine. In conclusion, we discovered a novel regulatory cascade that controls fungal nitrogen and carbon metabolism and is implicated in the entomopathogenicity of *M. robertsii*.

**IMPORTANCE** Penetration of the cuticle, the first physical barrier to pathogenic fungi, is the prerequisite for fungal infection of insects. In the entomopathogenic fungus Metarhizium robertsii, we found that the Fus3–mitogen-activated protein kinase (MAPK) and the transcription factor regulator of nutrient selection 1 (RNS1) constitute a novel cascade that controls cuticle penetration by regulating degradation of cuticular lipids, proteins, and chitin to obtain nutrients for hyphal growth and entry into the insect hemocoel. In addition, during saprophytic growth, the Fus3-MAPK/RNS1 cascade also activates utilization of complex and less-favored carbon and nitrogen sources that are not derived from insects. The homologs of Fus3-MAPK and RNS1 are widely found in ascomycete filamentous fungi, including saprophytes and pathogens with diverse hosts, suggesting that the regulation of utilization of nitrogen and carbon sources by the Fus3-MAPK/RNS1 cascade could be widespread. Our work provides significant insights into regulation of carbon and nitrogen metabolism in fungi and fungal pathogenesis in insects.

## INTRODUCTION

Fungi have evolved sophisticated regulatory mechanisms to enable them to respond rapidly to fluctuating environmental nutrient availability. Utilizing environmental carbon and nitrogen sources is a major physiological process to obtain the needed building blocks to sustain life and grow (1). Two regulatory mechanisms have been found to ensure that fungi preferentially utilize favored carbon (e.g., glucose) and nitrogen (e.g., ammonium [NH_4_^+^] and glutamine) sources and that fungi express genes for less-favored carbon or nitrogen source usage only in the absence of the favored nutrients (2). One pathway is the nitrogen metabolite repression (NMR). The protein NmrA deactivates the GATA transcription factor AREA by physical interaction in the presence of favored nitrogen, whereas in the absence of favored nitrogen sources, NAD^+^ binds to NmrA, which in turn dissociates from AREA, thereby allowing it to activate expression of the genes to utilize less-favored nitrogen sources (3). The other pathway is the carbon catabolite repression (CCR); via the repressor CreA, CCR ensures glucose is preferentially utilized by preventing the expression of genes for the metabolism of less-favored carbon sources (4–6). CCR and NMR converge on genes required for metabolizing several compounds that can be used as both carbon and nitrogen sources such as proline and arginine (7, 8). For example, in the plant-pathogenic fungus Magnaporthe oryzae, the trehalose-6-phosphate synthase Tps1 regulates NMR and CCR in response to glucose-6-phosphate (9). Although regulation of fungal nitrogen and carbon metabolism has been extensively investigated, the regulatory mechanisms remain to be fully understood, especially in multicellular fungal pathogens (9, 10).

The entomopathogenic fungus Metarhizium robertsii can switch among parasitic, saprophytic, and symbiotic lifestyles in response to changing environmental conditions because of its extremely versatile metabolism (11). Insect infection initiates when single-celled conidia adhere to the insect cuticle and produce multicellular hyphae, which tip cells differentiate into infection structures called appressoria. The fungus then penetrates the cuticle via mechanical pressure and cuticle-degrading enzymes. In the host hemocoel, the fungus proliferates as yeast-like hyphal bodies and kills the insects by a combination of fungal growth and toxins. Therefore, the insect cuticle is the first physical barrier to pathogenic fungi, and penetrating the cuticle is a prerequisite for fungal infection (12).

The insect cuticle is a nutrient-sparse environment, with lipids in the outermost layer and chitin and protein in the inner layer. Major signaling components, including mitogen-activated protein kinase (MAPK) cascades, Mr-OPY2, and cyclic adenosine 3′,5′-monophosphate protein kinase A (cAMP-PKA), and several transcription factors such as AFTF1 and MrSkn7 have been found to regulate cuticle penetration (13–15). These proteins control penetration of the cuticle by regulating genes for appressorial formation or for cuticle degradation. Proteases, chitinases, lipases, and P450s are responsible for degrading cuticular lipids, protein, and chitin, and the resulting products are used as nutrients for fungal growth (11, 16–18). However, how the upstream signaling pathways regulate the cuticle-degrading enzymes remains to be explored. The *Crr1* gene in CCR was described in *M. robertsii*, but its roles in utilization of cuticular nutrients are still unknown (19).

In our previous study, we reported that the Fus3-MAPK cascade positively regulates many cuticle-degrading genes (14). In this study, we found that regulation of cuticle degradation by Fus3-MAPK (named Fus3 in this paper for brevity) was mediated by the transcription factor RNS1. On the insect cuticle, Fus3 phosphorylates RNS1 to induce its own expression, which in turn activates the expression of cuticle-degrading genes. During saprophytic growth, the Fus3/RNS1 cascade also regulates utilization of complex less-favored carbon and nitrogen sources that are not derived from insects. Therefore, we discovered a novel cascade that regulates fungal carbon and nitrogen metabolism and is implicated in degradation of the insect cuticle to obtain nutrients for hyphal growth and entry into the hemocoel by the fungus *M. robertsii*.

## RESULTS

### RNS1 is regulated by Fus3 during cuticle penetration.

Our previous transcription sequencing (RNA-Seq) analysis showed that a transcription factor (XP_007823070) was positively regulated by Fus3 during cuticle penetration (14). XP_007823070 is designated RNS1, as it is a regulator of nutrient selection (see below). RNS1 is a Myb transcription factor containing a SANT domain (pfam00249). Homologs of RNS1 were identified in many other fungi, including the saprophytes Neurospora crassa (XP_960002) and Aspergillus ochraceoroseus (KKK12761), the insect pathogens Beauveria bassiana (XP_008593929) and Cordyceps militaris (XP_006673394), the plant pathogens *M. oryzae* (XP_003711780) and Fusarium graminearum (XP_011327324), and the mammal pathogens Histoplasma capsulatum (EEH11129) and Blastomyces dermatitidis (EQL37237).

**FIG 1:**
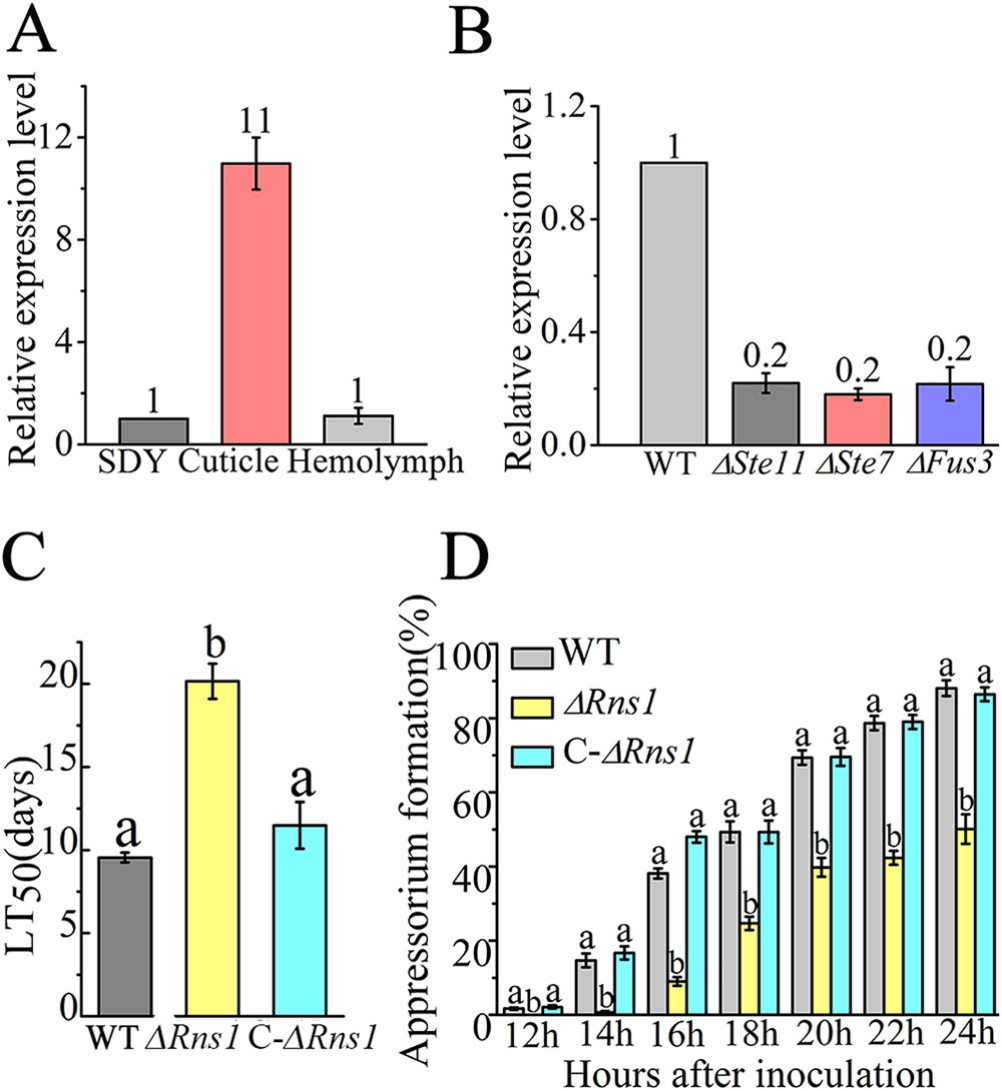
Expression pattern of *Rns1* and its involvement in cuticle penetration. (A) qRT-PCR analysis of *Rns1* expression during saprophytic growth (SDY), cuticle penetration (cuticle), and hemocoel colonization (hemolymph). All qRT-PCR analyses in this study were repeated three times, and the values in each figure represent the fold changes of expression of a gene in treatments compared with expression in their respective controls, which are set to 1. (B) qRT-PCR analysis of *Rns1* expression during cuticle penetration in the WT strain and the three deletion mutants in the Fus3-MAPK cascade: Δ*Fus3* (MAPK), Δ*Ste7* (MAPKK), and Δ*Ste11* (MAPKKK). (C) LT_50_ values when the insects were inoculated by topical application of conidia on the cuticle. Data are expressed as the means ± standard errors (SEs). Values with different lowercase letters are significantly different (*n* = 3, *P* < 0.05, Tukey’s test in one-way analysis of variance [ANOVA]). (D) Appressorial formation on a hydrophobic plastic surface. Within each time point, data with different lowercase letters are significantly different (*n* = 3, *P* < 0.05, Tukey’s test in one-way ANOVA). The experiment was repeated three times. Data are expressed as the means ± SEs. WT, wild-type strain; Δ*Rns1*, deletion mutant of *Rns1*; C-Δ*Rns1*, the complemented strain of the Δ*Rns1* mutant.

Quantitative reverse transcription-PCR (qRT-PCR) showed that *Rns1* was ∼11-fold more highly expressed during cuticle penetration (grown on the hindwings of Locusta migratoria locust adults) than during saprophytic growth (grown in the nutrient-rich medium Sabroud dextrose broth plus 1% yeast extract [SDY]) and hemocoel colonization (grown in the silkworm hemolymph), but no significant difference was found between saprophytic growth and hemocoel colonization (Fig. 1A). During cuticle penetration, *Rns1* was ∼5-fold more highly expressed in the wild-type (WT) strain than in the deletion mutants of the three kinase genes (Fus3-MAPK, Ste7-MAPKK, and Ste11-MAPKKK) in the Fus3-MAPK cascade (Fig. 1B), but no difference was found between the three mutants. During saprophytic growth and hemocoel colonization, no difference in *Rns1* expression was found between the WT strain and the Δ*Fus3* mutant (see Fig. S1A in the supplemental material).

10.1128/mSystems.00499-21.1 FIG S1 *Rns1* is not regulated by Fus3 during hemocoel colonization and saprophytic growth. (A) qRT-PCR analysis of *Rns1* expression during saprophytic growth in the SDY medium (SDY) and during hemocoel colonization (hemolymph). (B) LT_50_ values when the insects were inoculated by injection of conidia into the hemocoel. Data are expressed as the means ± SEs. Values with same lowercase letters are not significantly different (*n* = 3, *P > *0.05, Tukey’s test in one-way ANOVA). (C) Collapse rates of appressoria in the polyethylene glycol 8000 (PEG 8000) solution (80% [wt/vol]). The assays were repeated three times with three replicates per repeat. Data are expressed as the means ± SEs. Download FIG S1, TIF file, 0.7 MB. Copyright © 2021 Meng et al. 2021 Meng et al. https://creativecommons.org/licenses/by/4.0/ This content is distributed under the terms of the Creative Commons Attribution 4.0 International license.

To investigate the biological functions of *Rns1*, we constructed the deletion mutant of *Rns1* (Δ*Rns1*) and its complemented strain (C-Δ*Rns1*) (see Fig. S2A, B, and C). The genes and fungal strains used in this study are listed in the Table 1. Bioassays were conducted on Galleria mellonella larvae either by topically applying conidia to the insect cuticle or by direct injection of conidia into the hemocoel (thus bypassing the cuticle). Following topical application, the time taken to kill 50% of insects (LT_50_) of the Δ*Rns1* mutant (20.1 ± 1.1 days) was nearly 2-fold higher (*P* < 0.05) than the WT strain (10.2 ± 0.3 days), but no significant difference (*P* > 0.05) was found between the WT strain and the C-Δ*Rns1* strain (11.5 ± 1.4 days) (Fig. 1C). Via injection, no significant difference (*P* > 0.05) in LT_50_ was found among the WT, Δ*Rns1*, and C-Δ*Rns1* strains (Fig. S1B). On a normally inductive milieu (the hydrophobic surfaces of plastic petri dishes) in the presence of low levels of nitrogenous nutrients, compared with that in the WT strain, the appressorial formation of the Δ*Rns1* mutant was delayed, and again, no difference was found between the WT and C-Δ*Rns1* strains (Fig. 1D). No significant difference in appressorial turgor pressure was found between the WT, Δ*Rns1*, and C-Δ*Rns1* strains (Fig. S1C).

**TABLE 1:** Plasmids, fusion proteins, and fungal strains used in this study

Plasmid, protein, or strain	Description	Reference
Plasmids		
pPK2-Bar-Ptef-HA	Expression of a protein tagged with HA	25
pPK2-bar-Ptef-YFP^N^	Expression of a protein tagged with the N terminus of YFP	This study
pPK2-sur-Ptef-YFP^C^	Expression of a protein tagged with the C terminus of YFP	This study
pPK2-Sur-Ptef-FLAG	Expression of a protein tagged with FLAG	25
pPK2-Sur-Ptef-GFP-N	Expression of a protein tagged with GFP	25
Fusion proteins		
Fus3::HA	Fus3 tagged with HA	25
RNS1-DBD::YFP^N^	RNS1-DBD tagged with the N terminus of YFP	This study
Fus3::YFP^C^	Fus3 tagged with the C terminus of YFP	This study
RNS1::FLAG	RNS1 tagged with FLAG	This study
RNS1^T215A^::FLAG	Thr-215 replaced by alanine in RNS1::FLAG	This study
RNS1^S226A^::FLAG	Ser-226 replaced by alanine in RNS1::FLAG	This study
RNS1^T215A /S226A^::FLAG	Thr-215 and Ser-226 replaced by alanines in RNS1::FLAG	This study
RNS1-DBD	RNS1 portion (Glu-61 to Pro-267)	This study
RNS1-DBD::GFP	RNS1-DBD tagged with GFP	This study
RNS1^T215A^-DBD::GFP	Thr-215 replaced by alanine in RNS1-DBD::GFP	This study
RNS1^S226A^-DBD::GFP	Ser-226 replaced by alanine in RNS1-DBD::GFP	This study
Promoters		
*PRns1*	Promoter region (1,724 bp upstream of the ORF) of the gene *Rns1*	This study
*PRns1*^Δ^*^BM2^*	Mutated *PRns1* with all 7 nt in the motif *BM2* changed to As	This study
Genomic clones		
*gRns1*	Genomic clone of the *Rns1* gene	This study
*gRns1^T215A^*	*gRns1* mutated to replace Thr-215 with alanine in RNS1 protein	This study
*gRns1^S226A^*	*gRns1* mutated to replace Ser-226 with alanine in RNS1 protein	This study
*gRns1*^Δ^*^BM2^*	*gRns1* with 7 nt of *BM2* changed to As in the promoter *PRns1*	This study
Fungal strains		
WT	Wild-type strain of *M. robertsii* ARSEF 2575	This study
Δ*Rns1*	Deletion mutant of the *Rns1* gene	This study
C-Δ*Rns1*	Complemented strain of the Δ*Rns1* mutant	This study
*WT-FLAG*	Expressing a FLAG tag in the WT strain	This study
*WT-RNS1-FLAG*	Expressing RNS1::FLAG in the WT strain	This study
*WT-RNS1^T215A^-FLAG*	Expressing RNS1^T215A^::FLAG in the WT strain	This study
*WT-RNS1^S226A^-FLAG*	Expressing RNS1^S226A^::FLAG in the WT strain	This study
*WT-RNS1^T215A/S226A^-FLAG*	Expressing RNS1^T215A/S226A^::FLAG in the WT strain	This study
Δ*Fus3-RNS1-FLAG*	Expressing RNS1::FLAG in the Δ*Fus3* mutant	This study
Δ*Fus3-RNS1^T215A^-FLAG*	Expressing RNS1^T215A^::FLAG in the Δ*Fus3* mutant	This study
Δ*Fus3-RNS1^S226A^-FLAG*	Expressing RNS1^S226A^::FLAG in thet Δ*Fus3* mutan	This study
Δ*Fus3-RNS1^T215A/S226A^-FLAG*	Expressing RNS1^T215A/S226A^::FLAG in the WT strain	This study
Δ*Rns1-RNS1-FLAG*	Expressing RNS1::FLAG in the Δ*Rns1* mutant	This study
Δ*Rns1-FLAG*	Expressing a FLAG tag in the Δ*Rns1* mutant	This study
*RNS1-FLAG/Fus3-HA*	Expressing RNS1::FLAG and Fus3::HA in the WT strain	This study
*RNS1-DBD-YFP^N^/Fus3-YFP^C^*	Expressing RNS1-DBD::YFP^N^ and Fus3::YFP^C^ in the WT strain	This study
*RNS1-DBD-YFP^N^/YFP^C^*	Expressing RNS1-DBD::YFP^N^ and YFP^C^ in the WT strain	This study
*WT-RNS1-DBD-GFP*	Expressing RNS1-DBD::GFP in the WT strain	This study
*WT-RNS1^T215A^-DBD-GFP*	Expressing RNS1^T215A^-DBD::GFP in the WT strain	This study
*WT-RNS1^S226A^-DBD-GFP*	Expressing RNS1^S226A^-DBD::GFP in the WT strain	This study
Δ*Fus3-RNS1-DBD-GFP*	Expressing RNS1-DBD::GFP in the Δ*Fus3* mutant	This study
Δ*Fus3-RNS1^T215A^-DBD-GFP*	Expressing RNS1^T215A^-DBD::GFP in the Δ*Fus3* mutant	This study
Δ*Fus3-RNS1^S226A^-DBD-GFP*	Expressing RNS1^S226A^-DBD::GFP in the Δ*Fus3* mutant	This study
Δ*Rns1-gRns1^T215A^*	Δ*Rns1* mutant transformed with the genomic clone *gRns1^T215A^*	This study
Δ*Rns1-gRns1^S226A^*	Δ*Rns1* mutant transformed with the genomic clone *gRns1^S226A^*	This study
Δ*Rns1-gRns1*^Δ^*^BM2^*	Δ*Rns1* mutant transformed with the genomic clone *gRns1*^Δ^*^BM2^*	This study
*WT-PRns1-gfp*	*gfp* gene driven by the promoter *PRns1* in the WT strain	This study
*WT-PRns1*^Δ^*^BM2^-gfp*	*gfp* gene driven by the mutant promoter *PRns1*^Δ^*^BM2^* in the WT strain	This study
Δ*Rns1-PRns1-gfp*	*gfp* gene driven by the promoter *PRns1* in theΔ*Rns1* mutant	This study
Δ*Rns1-PRns1*^Δ^*^BM2^-gfp*	*gfp* gene driven by the mutant promoter *PRns1*^Δ^*^BM2^* in the Δ*Rns1* mutant	This study
Δ*Fus3-PRns1-gfp*	*gfp* gene driven by the promoter *PRns1* in the Δ*Fus3* mutant	This study
Δ*Fus3-PRns1*^Δ^*^BM2^-gfp*	*gfp* gene driven by the mutant promoter *PRns1*^Δ^*^BM2^* in the Δ*Fus3* mutant	This study

10.1128/mSystems.00499-21.2 FIG S2 Confirmation of gene deletion and knockdown. (A) Schematic diagram of deleting a gene based on homologous recombination. Map of a deletion plasmid (bottom) and its relative position in the fungal genome (top). (B) Confirmation of the deletion of *Rns1* and complementation of the mutant with PCR. D1 and D2, two independent deletion mutants; WT, wild-type strain; M, DNA ladder. (Top) PCR performed with primers CF1/CF2 (the relative positions of all primers are shown in panel A); PCR products were obtained only in the WT strain. (Middle) PCR with Bar-up/CF2; PCR products were obtained only from the gene deletion mutants. (Bottom) Confirmation of the complementation of the Δ*Rns1* deletion mutants using PCR. C, complemented strain; D, Δ*Rns1* mutant. (C) Confirmation of insertion of the mutants of the genomic clone *gRns1* into the Δ*Rns1* mutant. 1, Δ*Rns1-gRns1^T215A^* mutant; 2, Δ*Rns1-gRns1^S226A^* mutant; 3, Δ*Rns1-gRns1*^Δ^*^BM2^* mutant; D, Δ*Rns1* mutant; WT, wild-type strain. Confirmation of the deletion of *AreA* (D), *Crr1* (E), and *Snf1* (F) with PCR. (Top) PCR with primers CF1/CF2; (bottom) PCR with Bar-up/CF2. (G) qRT-PCR analysis of *Tor, Tps1*, and *G6PD* in the WT strain and their knockdown mutants. #1, #2, and #3, three independent knockdown isolates. Download FIG S2, TIF file, 2.9 MB. Copyright © 2021 Meng et al. 2021 Meng et al. https://creativecommons.org/licenses/by/4.0/ This content is distributed under the terms of the Creative Commons Attribution 4.0 International license.

### Fus3 phosphorylates RNS1 during cuticle penetration.

**FIG 2:**
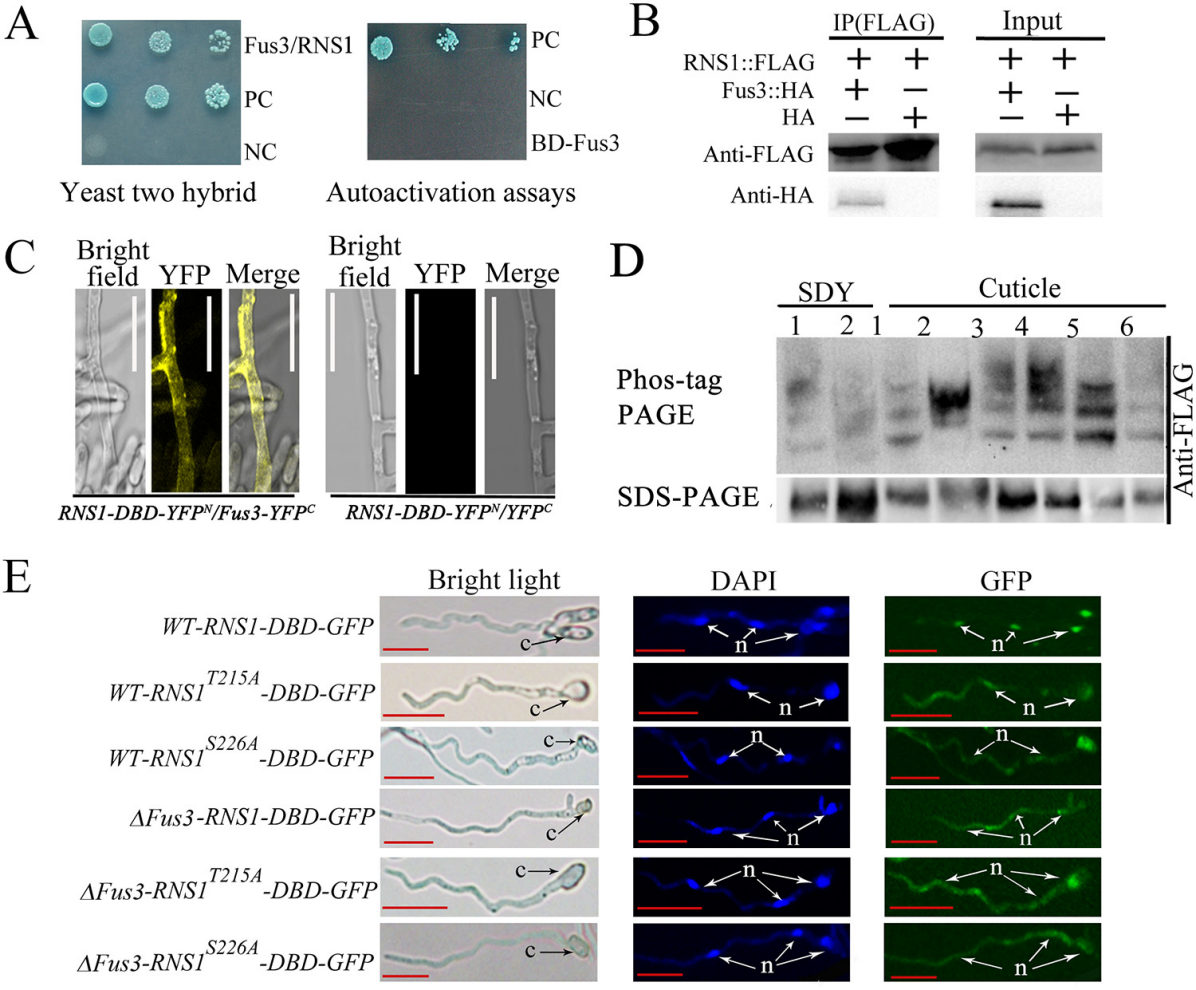
Phosphorylation of RNS1 by Fus3 is important for its entry into the nucleus. (A) Yeast two-hybrid analysis showing Fus3 interacts with RNS1. (Left) Colonies grown in SD-His-Ade-Leu-Trp plus X-α-Gal plus AbA. Fus3/RNS1, cells expressing Fus3 and RNS1. (Right) Colonies grown in SD-His-Trp-Ade plus X-α-Gal. BD-Fus3, Y2HGold cells expressing Fus3; NC, negative control; PC, positive control. (B) Co-IP confirmation of the interaction of Fus3 with RNS1. Immunoprecipitation was conducted with anti-FLAG antibody. Proteins were detected by immunoblot analysis with anti-HA or anti-FLAG antibodies. (C) BiFC analysis showing Fus3 interacted with RNS1. (Left) Strain expressing RNS1-DBD-YFP^N^ and Fus3-YFP^C^. (Right) Strain expressing RNS1-DBD-YFP^N^ and YFP^C^. Bars, 20 μm. (D) Phos-tag analysis of RNS1 phosphorylation by Fus3. 1, Δ*Fus3-RNS1-FLAG* strain; 2, *WT-RNS1-FLAG* strain; 3, *WT-RNS1^T215A^-FLAG* strain; 4, Δ*Fus3-RNS1^T215A^-FLAG* strain; 5, *WT-RNS1^S226A^-FLAG* strain; 6, Δ*Fus3-RNS1^S226A^-FLAG* strain; SDY, SDY medium; cuticle, cuticle medium. (E) Phosphorylation of the sites Thr-215 and Ser-226 by Fus3 is important for RNS1 entry into the nucleus. The strain names are shown on the left. DAPI, 4′,6-diamidino-2-phenylindole; c, conidium; n, nucleus. Bars, 5 μm. Detailed description of the strains is shown in Table 1. In this study, all shown images are representative of at least three independent experiments.

To investigate how Fus3 regulates *Rns1* expression, we first assayed whether Fus3 physically contacted RNS1. Yeast two-hybrid assays showed that Fus3 physically interacted with RNS1 (Fig. 2A). Using an *RNS1-FLAG/Fus3-HA* strain that expressed RNS1::FLAG (a protein with the FLAG fused to RNS1) and Fus3::HA (a protein with Fus3 tagged with hemagglutinin), a coimmunoprecipitation (Co-IP) assay confirmed that RNS1 physically interacted with Fus3 *in vivo* (Fig. 2B). Unless otherwise indicated, all fusion proteins are driven by the constitutive promoter *Ptef* from Aureobasidium pullulans in this study. For a biomolecular fluorescence complementation (BiFC) assay, construction of a strain expressing a protein with RNS1 fused with the N terminus of YFP (YFP^N^) failed, but we successfully constructed a protein RNS1-DBD::YFP^N^ with YFP^N^ fused to a portion of RNS1 (named RNS1-DBD, Glu-61 to Pro-267) containing the nuclear localization signal (NLS), DNA binding domain (DBD), and Fus3 phosphorylation sites (see below). A clear YFP signal was detected in the cells of the *RNS1-DBD-YFP^N^/Fus3-YFP^C^* strain that expressed the fusion proteins RNS1-DBD::YFP^N^ and Fus3::YFP^C^ (Fig. 2C). YFP^C^ is the C terminus of YFP. YFP signal was not observed in the negative-control *RNS1-DBD-YFP^N^/YFP^C^* strain expressing RNS1-DBD::YFP^N^ and YFP^C^ (Fig. 2C).

To investigate whether Fus3 phosphorylates RNS1, the *WT-RNS1-FLAG* and Δ*Fus3-RNS1-FLAG* strains were constructed by expressing RNS1::FLAG in the WT strain and the Δ*Fus3* mutant, respectively (see Fig. S3A). RNS1 phosphorylation was assayed with the Phos-tag method. Since it was not possible to obtain enough biomass to get sufficient protein for this assay by growing *M. robertsii* on the locust hindwings, we cultivated the fungus in the cuticle medium with the insect cuticle as a sole carbon and nitrogen sources to approximate cuticle penetration. In the cuticle medium, compared with that of the Δ*Fus3-RNS1-FLAG* strain, the band of the RNS1::FLAG protein shifted in the *WT-RNS1-FLAG* strain (Fig. 2D). When the *WT-RNS1-FLAG* strain was transferred from the SDY medium to the cuticle medium, this band shift was also seen, but no obvious difference in the positions of the RNS1::FLAG protein was seen between the *WT-RNS1-FLAG* strain in the SDY medium and the Δ*Fus3-RNS1-FLAG* strain in the cuticle medium or the SDY medium (Fig. 2D).

10.1128/mSystems.00499-21.3 FIG S3 Confirmation of expression of fusion proteins. (A) Expression of the fusion proteins RNS1::FLAG, RNS1^T215A^::FLAG or RNS1^S226A^::FLAG in the WT strain or in the Δ*Fus3* mutant. 1, *WT-RNS1-FLAG*; mutant; 2, Δ*Fus3-RNS1-FLAG* mutant; 3, *WT-RNS1^T215A^-FLAG* mutant; 4, Δ*Fus3-RNS1^T215A^-FLAG* mutant; 5, *WT-RNS1^S226A^-FLAG* mutant; 6, Δ*Fus3-RNS1^S226A^-FLAG* mutant; 7, Δ*Rns1-RNS1-FLAG* mutant; 8, *WT-FLAG* strain. Note that the band corresponding to the FLAG tag was not seen due to its small size. M, protein ladder (Thermo Fisher Scientific). (B) qRT-PCR analysis of the RNS1::FLAG-encoding gene in the *WT-RNS1-FLAG*, Δ*Fus3-RNS1-FLAG*, *WT-RNS1^T215A/S226A^-FLAG*, and Δ*Fus3-RNS1^T215A/S226A^-FLAG* strains. Two randomly selected isolates of the *WT-RNS1^T215A/S226A^-FLAG* and Δ*Fus3-RNS1^T215A/S226A^-FLAG* strains were analyzed. (C) Expression of RNS1::FLAG and RNS1^T215A/S226A^::FLAG. 1, *WT-RNS1-FLAG* mutant; 2, Δ*Fus3-RNS1-FLAG* mutant; 3 and 4, two isolates of the *WT-RNS1^T215A/S226A^-FLAG* strain; 5 and 6, two isolates of the Δ*Fus3-RNS1^T215A/S226A^-FLAG* strain. (D) Impact of the proteasome inhibitor MG132 (0.2 mM) and the autophagy inhibitor 3-methyladenine (3-MA; 0.2 mM) treatment on the expression of RNS1^T215A/S226A^::FLAG in the *WT-RNS1^T215A/S226A^-FLAG* strain. 1, not treated with inhibitors; 2, treatment with MG132 for 4 h; 3, treatment with MG132 for 8 h; 4, treatment with 3-MA for 4 h; 5, treatment with 3-MA for 8 h. Note that the protein RNS1^T215A/S226A^::FLAG was not detected. (E) Expression of the proteins RNS1-DBD::GFP, RNS1^T215A^-DBD::GFP, and RNS1^S226A^-DBD::GFP in the WT strain or in the Δ*Fus3* mutant. 1*, WT-RNS1-DBD-GFP* mutant; 2, Δ*Fus3-RNS1-DBD-GFP* mutant; 3, *WT-RNS1^T215A^-DBD-GFP* mutant; 4, Δ*Fus3-RNS1^T215A^-DBD-GFP*; mutant 5, *WT-RNS1^S226A^-DBD-GFP* mutant; 6, Δ*Fus3-RNS1^S226A^-DBD-GFP* mutant. Detailed description of the strains is shown in Table 1. In this study, all shown images are representative of at least three independent experiments. Download FIG S3, TIF file, 0.8 MB. Copyright © 2021 Meng et al. 2021 Meng et al. https://creativecommons.org/licenses/by/4.0/ This content is distributed under the terms of the Creative Commons Attribution 4.0 International license.

According to the characteristics of the serine/threonine amino acids targeted by MAPK kinases (20), the threonine at position 215 (Thr-215) and the serine at position 226 (Ser-226) in RNS1 were predicted to be Fus3 phosphorylation sites. To confirm these predictions, we constructed two RNS1 mutants (RNS1^T215A^::FLAG or RNS1^S226A^::FLAG) with Thr-215 or Ser-226 in the protein RNS1::FLAG, respectively, replaced with alanine. They were expressed in the WT strain to produce the strains *WT-RNS1^T215A^-FLAG* and *WT-RNS1^S226A^-FLAG* and in the Δ*Fus3* mutant to generate Δ*Fus3-RNS1^T215A^-FLAG* and Δ*Fus3-RNS1^S226A^-FLAG* strains (Fig. S3A). In the cuticle medium, comparison of *WT-RNS1-FLAG*, *WT-RNS1^S226A^-FLAG*, and *WT-RNS1^T215A^-FLAG* strains showed that the mutations of Thr-215 and Ser-226 significantly affected RNS1 phosphorylation (Fig. 2D). Three bands were seen in *WT-RNS1^T215A^-FLAG* and *WT-RNS1^S226A^-FLAG* strains, and two of them had the same migration speed, indicating that the differential bands between the two strains corresponded to the proteins with Thr-215 or Ser-226 phosphorylated. But the difference in migration of these two differential bands was not seen in Δ*Fus3-RNS1^T215A^-FLAG* and Δ*Fus3-RNS1^S226A^-FLAG* strains.

We also constructed a *WT-RNS1^T215A/S226A^-FLAG* strain expressing the protein RNS1^T215A/S226A^::FLAG with both Thr-215 and Ser-226 replaced by alanines. *RNS1^T215A/S226A^-FLAG* was constitutively transcribed, but its protein was not detectable, which was not attributed to the proteasome pathway and autophagy (Fig. S3B to D). Therefore, phosphorylation of Thr-215 and Ser-226 could not be simultaneously assayed.

### Fus3-mediated phosphorylation facilitates RNS1 entry into nuclei.

To investigate the impact of Fus3-mediated phosphorylation on RNS1 entry into nuclei, we constructed three green fluorescent protein (GFP)-tagged proteins: RNS1-DBD::GFP, RNS1^T215A^-DBD::GFP, and RNS1^S226A^-DBD::GFP, which were then expressed in the WT strain to produce *WT-RNS1-DBD-GFP*, *WT-RNS1^T215A^-DBD-GFP*, and *WT-RNS1^S226A^-DBD-GFP* strains, respectively, and in the Δ*Fus3* mutant to form Δ*Fus3-RNS1-DBD-GFP*, Δ*Fus3-RNS1^T215A^-DBD-GFP*, and Δ*Fus3-RNS1^S226A^-DBD-GFP* strains, respectively (Fig. S3E). On the insect cuticle, the GFP signal was concentrated in the nuclei of the *WT-RNS1-DBD-GFP* strain, but it was almost evenly distributed in the cells of all five other strains (Fig. 2E).

### Identification of the DNA motifs recognized by RNS1.

Chromatin immunoprecipitation sequencing (ChIP-Seq) analysis was conducted to identify the DNA motif recognized by RNS1. To this end, the Δ*Rns1-RNS1-FLAG* strain was constructed with RNS1::FLAG expressed in the Δ*Rns1* mutant (Fig. S3A). A total of 3, 616 peaks were identified (see Fig. S4A), and six consensus motifs were present with a high confidence score (Fig. S4B).

10.1128/mSystems.00499-21.4 FIG S4 Identification of DNA motifs recognized by RNS1. (A) ChIP-Seq analysis of genome-wide RNS1 binding motifs. Shown is the distribution of all RNS1 peak locations within the genome. (B) Top six motifs enriched by HOMER software. (C) SDS-PAGE analysis of the expression and purification of the recombinant protein RNS1-DBD in E. coli. M, protein ladder; 1, crude extract from the cells with the plasmid pET-28a-SUMO (control); 2, crude extract from the cells expressing RNS1-DBD; 3, supernatant of the crude extract shown in lane 2; 4, proteins purified from the supernatant shown in lane 3 with the HisPur Ni-NTA resin; 5, proteins after the protease ULP1 treatment with the proteins from lane 4 to remove the SUMO tag; 6, homogenous RNS1-DBD purified from the proteins of lane 5 with the HisPur Ni-NTA resin. (D) Western blot analysis confirming expression of the protein SUMO::RNS1-DBD using the anti-His tag antibody. 1 and 2 are proteins from lane 3 and 4, respectively, described for panel C. EMSAs of the *in vitro* binding of RNS1-DBD to motif 3 (E), motif 4 (F), motif 5 (G), and motif 6 (H). Note that the shift of the labeled DNA was not seen with these four DNA motifs. The names of the genes whose promoters contain a motif are shown at the bottom. (I) EMSA of the importance of the nucleotides in the *BM1* motif in its binding to RNS1-DBD. (Top) Sequences of the DNA probes containing the *BM1* motif (10 nucleotides shown in bold) within the promoter of the gene MAA_10686. The name of the DNA probe is shown in the left, and its sequence is in the right. BM1, the WT DNA probe; M1, the nucleotide at position 1 in the *BM1* motif is mutated into a nucleotide shown in bold lowercase letter. The naming system is also used for all other mutated DNA probes. (Bottom) EMSA of the binding of the DNA probe to RNS1-DBD. The binding activity was demonstrated by the labeled DNA band shift prior to the addition of the specific competitor (the unlabeled WT DNA probe) in a 200-fold excess. The importance of each nucleotide in the *BM1* motif in the binding of the DNA probe to RNS1-DBD is shown by the impact of the addition of an unlabeled mutated probe as a competitor in a 200-fold excess on the labeled DNA band shift. The names of the unlabeled mutated DNA probes are shown above their respective lanes. (J) Importance of the nucleotides in the *BM2* motif in its binding to RNS1-DBD. The legends for the top and bottom panels are the same as described for panel I for the *BM1* motif. The DNA probe containing the *BM2* motif is within the promoter of the gene MAA_05782. Download FIG S4, TIF file, 2.9 MB. Copyright © 2021 Meng et al. 2021 Meng et al. https://creativecommons.org/licenses/by/4.0/ This content is distributed under the terms of the Creative Commons Attribution 4.0 International license.

**FIG 3:**
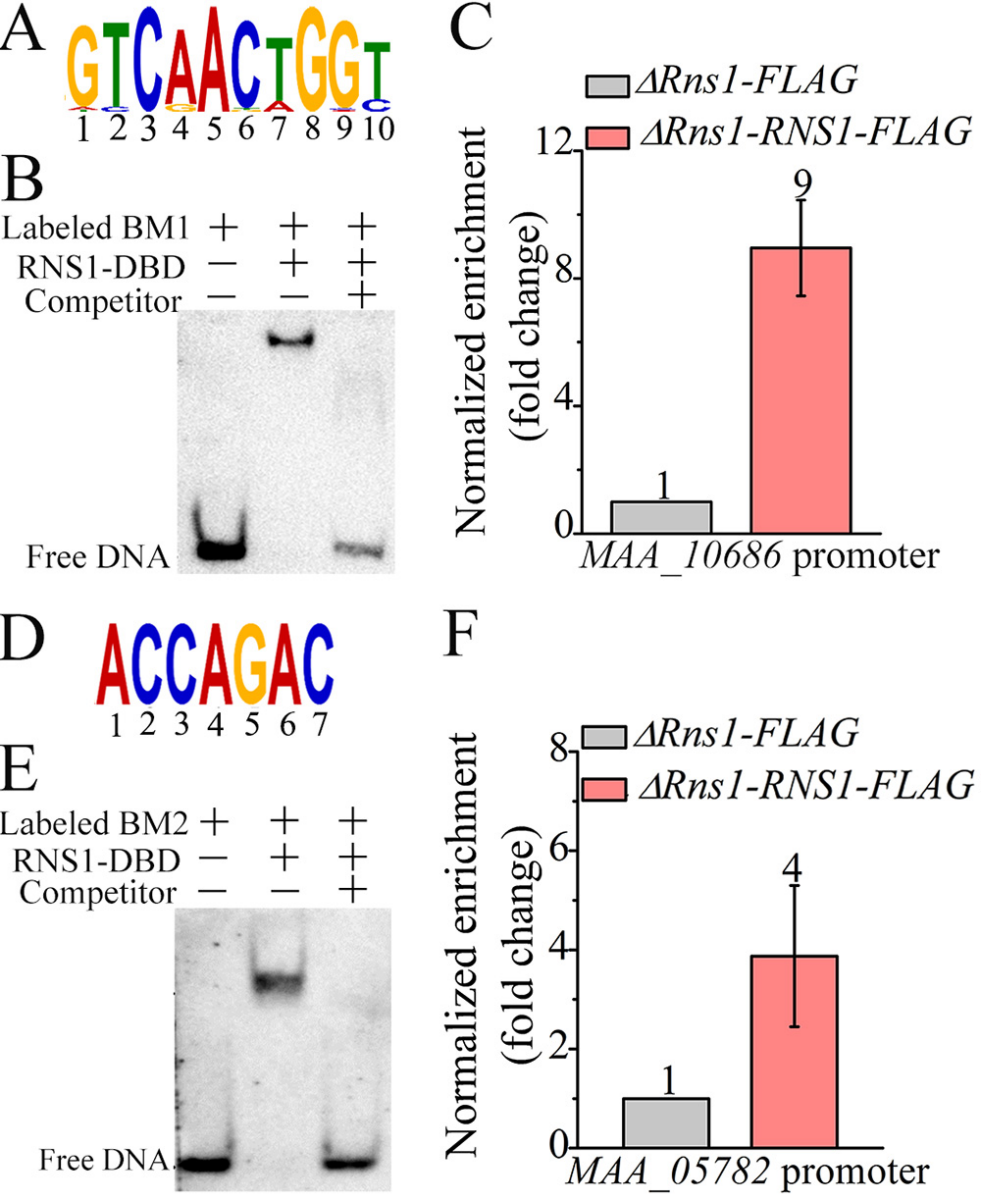
Identification of the DNA motifs bound by RNS1. (A) Consensus DNA motif *BM1*. (B) EMSA confirmed the *in vitro* binding of the biotin-labeled *BM1* to the recombinant protein RNS1-DBD. The binding activity was demonstrated by the shift of the labeled DNA band prior to the addition of the specific competitor (the unlabeled DNA probe) in a 200-fold excess. The tested motif *BM1* is in the promoter of the gene MAA_10686. (C) ChIP-qPCR analysis confirms that RNS1 *in vivo* binds to the motif *BM1* in the MAA_10686 promoter. Detailed description of the Δ*Rns1-RNS1-FLAG* and Δ*Rns1-FLAG* strains is shown in Table 1. (D) Consensus DNA motif *BM2*. (E) EMSA confirmed the *in vitro* binding of the biotin-labeled *BM2* to the protein RNS1-DBD. The tested motif *BM2* is in the promoter of the gene MAA_05782. (F) ChIP-qPCR analysis shows that RNS1 *in vivo* binds to the motif *BM2* in the MAA_05782 promoter.

For electrophoretic mobility shift assays (EMSAs), we failed to express the whole protein of RNS1 in Escherichia coli. However, RNS1-DBD was successfully expressed and purified (Fig. S4C and D) for assaying the binding of RNS1-DBD to six biotin-labeled DNA probes, each containing one of the six consensus motifs (Fig. S4E to H). The recombinant RNS1-DBD protein bound to two DNA probes, which contained the 10-nucleotide (nt) motif *BM1* [G(A/T)T(C)CA(G)AC(T/G)T(A)GG(C/A)T(C)] and the 7-nt motif *BM2* (ACCAGAC) (Fig. 3A and D). For these two DNA probes, their respective specific competitor (unlabeled DNA probe) abolished the DNA band shift (Fig. 3B and E). Chromatin immunoprecipitation-quantitative PCR (ChIP-qPCR) analysis showed that the copy number of the enriched DNA fragment containing the *BM1* motif in the promoter of the gene MAA_10686 from the Δ*Rns1-RNS1-FLAG* strain was 9-fold higher than in the Δ*Rns1-FLAG* strain that expressed the FLAG tag (Fig. 3C). For the motif *BM2* in the promoter of the gene MAA_05782, the copy number of the enriched DNA fragment from the Δ*Rns1-RNS1-FLAG* strain was 4-fold higher than in the Δ*Rns1-FLAG* strain (Fig. 3F).

To assay the importance of the 10 nucleotides of the *BM1* motif in its binding to RNS1-DBD, unlabeled *BM1* mutants were constructed by replacing a consensus nucleotide with each of other three, which were subsequently used as competitors for the binding of RNS1-DBD to the biotin-labeled WT DNA probe. None of the mutations in the 10 consensus nucleotides completely abolished the shift of the band of the biotin-labeled WT DNA probe, but the mutations of the nucleotides at positions 8 and 9 had the least impact on the band shift (Fig. S4I). Similar experiments were also conducted for the motif *BM2*, and mutations of the nucleotides at positions 5 and 7 had no impact on the band shift of the biotin-labeled WT DNA probe, but all other mutated DNA probes completely abolished the band shift (Fig. S4J).

### Phosphorylated RNS1 upregulates its own expression.

**FIG 4:**
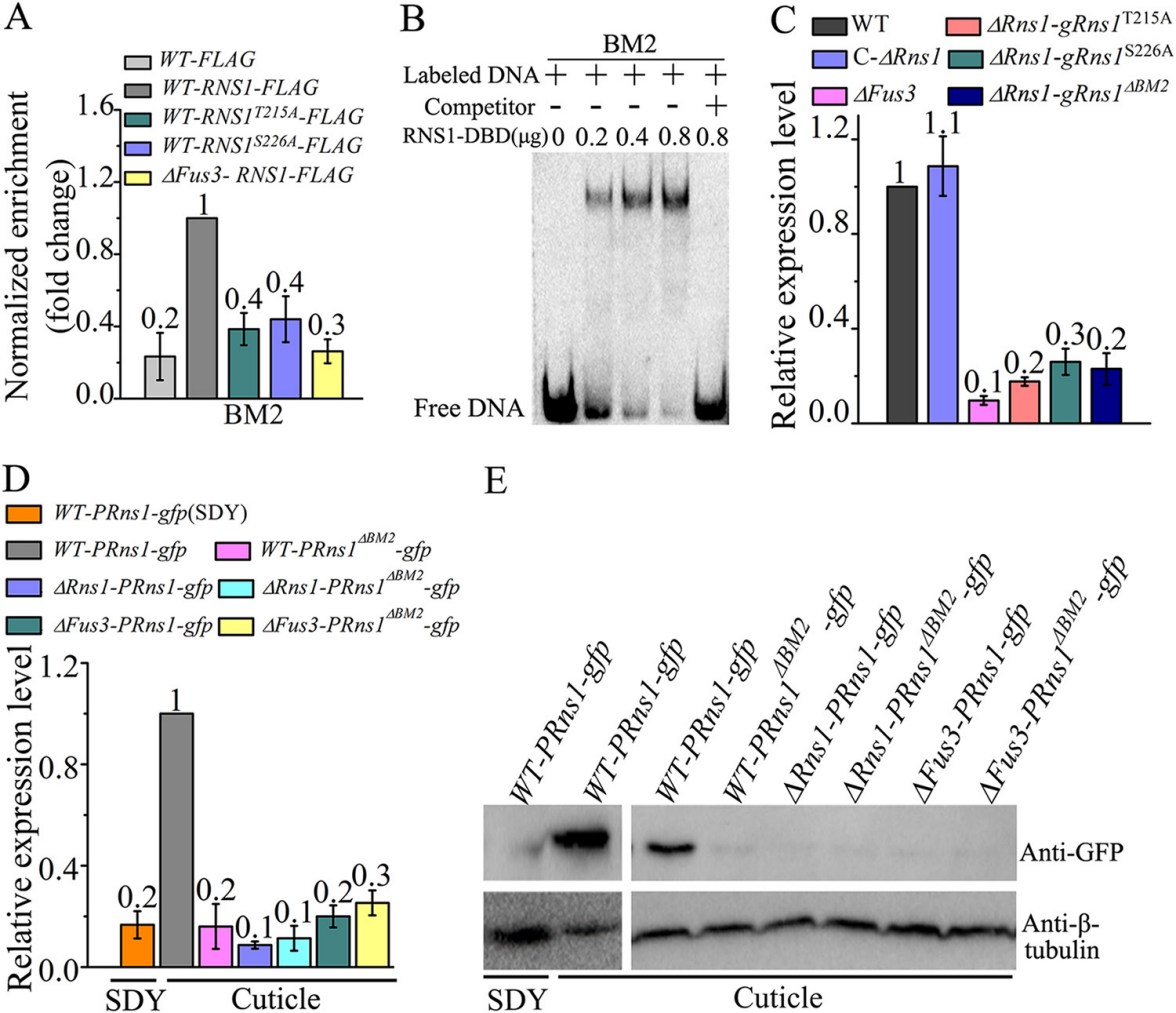
Phosphorylation of RNS1 by Fus3 facilitates binding to its own promoter to self-induce expression during cuticle penetration. (A) ChIP-qPCR analysis of *in vivo* binding of RNS1 to the *BM2* motif in its own promoter during cuticle penetration. Detailed description of the strains in this figure is shown in Table 1. (B) EMSA confirmed *in vitro* binding of the recombinant protein RNS1-DBD to the biotin-labeled *BM2* motif in the *Rns1* promoter. The specific competitor (unlabeled DNA motifs) was added in a 200-fold excess. (C) qRT-PCR analysis of *Rns1* expression in the WT strain, the Δ*Fus3* mutant, and the strains obtained by transforming the Δ*Rns1* mutant with the wild-type genomic clone *gRns1* or mutants of *gRns1.* qRT-PCR analysis (D) and Western blot analysis (E) of the expression of GFP in the strains with *gfp* driven by the wild-type *Rns1* promoter *PRns1* or its mutant *PRns1*^Δ^*^BM2^* (*PRns1* with the motif *BM2* mutated) in cuticle medium (cuticle) or SDY medium (SDY).

We then investigated how *Rns1* was upregulated during cuticle penetration. A *BM1* motif (GTCGACTGGC) and a *BM2* motif (CCCAGAC) were found in the *Rns1* promoter. During the cuticle penetration, ChIP-qPCR analysis showed that a higher copy number of the *BM2* motif was enriched from the *WT-RNS1-FLAG* strain than from the *WT-FLAG* strain that expressed the FLAG (Fig. 4A). EMSA also showed that the recombinant RNS1-DBD protein bound to the *BM2* motif (Fig. 4B). ChIP-qPCR analysis showed that RNS1 did not bind to the *BM1* motif, though EMSA showed that RNS1-DBD *in vitro* weakly bound to this motif (see Fig. S5A and B).

10.1128/mSystems.00499-21.5 FIG S5 RNS1 did not bind to the *BM1* but *BM2* motif during cuticle penetration. (A) EMSA confirmed *in vitro* binding of the recombinant protein RNS1-DBD to the biotin-labeled *BM1* motif in the *Rns1* promoter. The specific competitor (unlabeled DNA motifs) was added in a 200-fold excess. (B) ChIP-qPCR analysis of the binding of the fusion proteins RNS1::FLAG, RNS1^T215A^::FLAG, and RNS1^S226A^::FLAG to the *BM1* motif in the *Rns1* promoter in different strains during cuticle penetration. Note that RNS1 did not bind to the putative *BM1* motif in its own promoter. (C) qRT-PCR analysis of the transcription of the genes encoding the fusion proteins RNS1::FLAG, RNS1^T215A^::FLAG, and RNS1^S226A^::FLAG in different strains. (D) ChIP-qPCR analysis of the binding of the protein RNS1::FLAG to the *BM2* motif in the *Pr1a* (MAA_05675) and *Pr1b* (MAA_08168) promoters during cuticle penetration. Note that RNS1 bound to the *BM2* motif. (E) ChIP-qPCR analysis of the binding of the fusion proteins RNS1::FLAG to the *BM1* motif in the MAA_10686 promoter during cuticle penetration. Note that RNS1 did not bind to the *BM1* motif. Detailed description of the strains is shown in Table 1. Download FIG S5, TIF file, 2.9 MB. Copyright © 2021 Meng et al. 2021 Meng et al. https://creativecommons.org/licenses/by/4.0/ This content is distributed under the terms of the Creative Commons Attribution 4.0 International license.

To assay the impact of the Fus3-mediated phosphorylation on the binding of RNS1 to the *BM2* motif in the *Rns1* promoter, we constructed three strains: *WT-RNS1^T215A^-FLAG* expressing the protein RNS1^T215A^::FLAG, *WT-RNS1^S226A^-FLAG* expressing RNS1^S226A^::FLAG, and Δ*Fus3-RNS1-FLAG* with RNS1::FLAG expressed in the Δ*Fus3* mutant (Fig. S3A). During cuticle penetration, no difference in the transcription level of the fusion genes was found among the *WT-RNS1-FLAG*, *WT-RNS1^T215A^-FLAG*, *WT-RNS1^S226A^-FLAG*, and Δ*Fus3-RNS1-FLAG* strains (Fig. S5C). ChIP-qPCR analysis showed that a higher copy number of the *BM2* motif was enriched in the *WT-RNS1-FLAG* strain than in the *WT-RNS1^T215A^-FLAG*, *WT-RNS1^S226A^-FLAG*, and Δ*Fus3-RNS1-FLAG* strains, but no significant difference was found between the latter three strains (Fig. 4A).

To investigated the impact of the binding of RNS1 to its own promoter on its expression, we constructed three mutated genomic clones of *gRns1* (used for complementing the Δ*Rns1* mutant as described above): *gRns1*^Δ^*^BM2^* by changing all consensus nucleotides in the motif *BM2* to adenine, *gRns1^T251A^* by substituting alanine for Thr-215, and *gRns1^S226A^* by replacing Ser-226 with alanine. *gRns1*^Δ^*^BM2^*, *gRns1^T215A^*, and *gRns1^S226A^* were then transformed into the Δ*Rns1* mutant to produce Δ*Rns1-gRns1*^Δ^*^BM2^*, Δ*Rns1-gRns1^T215A^*, and Δ*Rns1-gRns1^S226A^* strains, respectively (Fig. S2C). During cuticle penetration, qRT-PCR showed that *Rns1* was more highly expressed in the WT strain than in the Δ*Rns1-gRns1*^Δ^*^BM2^*, Δ*Rns1-gRns1^T215A^*, Δ*Rns1-gRns1^S226A^*, and Δ*Fus3* strains, and no significant difference was found between the latter four strains. The C-Δ*Rns1* strain had the same transcription level of *Rns1* as the WT strain (Fig. 4C).

Another assay was conducted to confirm that *Rns1* self-induces expression. To this end, we constructed two *gfp* gene cassettes: *PRns1-gfp* with *gfp* driven by *Rns1* promoter *PRns1*, and *PRns1*^Δ^*^BM2^-gfp* with *gfp* driven by the promoter *PRns1*^Δ^*^BM2^* with the motif *BM2* mutated in *PRns1*. These two cassettes were transformed into the WT strain to produce *WT-PRns1-gfp* and *WT-Pns1*^Δ^*^BM2^-gfp* strains, into the Δ*Rns1* mutant to generate Δ*Rns1-PRns1-gfp* and Δ*Rns1-PRns1*^Δ^*^BM2^-gfp* strains, and into the Δ*Fus3* mutant to form Δ*Fus3-PRns1-gfp* and Δ*Fus3-PRns1*^Δ^*^BM2^-gfp* strains. For all strains, two independent isolates showed the same results, and so only one of them is presented here. For the *WT-PRns1-gfp* strain, compared with that during saprophytic growth, the expression levels of GFP transcript and protein were upregulated during cuticle penetration (Fig. 4D and E), showing that the *Rns1* promoter *PRns1* retains its activity in its nonnative chromosomal positions. During cuticle penetration, GFP transcript and protein were more highly expressed in the *WT-PRns1-gfp* strain than in the *WT-PRns1*^Δ^*^BM2^-gfp*, Δ*Rns1-PRns1-gfp*, Δ*Rns1-PRns1*^Δ^*^BM2^-gfp*, Δ*Fus3-PRns1-gfp*, and Δ*Fus3-PRns1*^Δ^*^BM2^-gfp* strains, whereas no differences were found between the latter five strains (Fig. 4D and E).

### RNS1 induces cuticle-degrading genes.

RNA-Seq analysis was used to profile genes regulated by RNS1 during cuticle penetration. Compared with expression in the WT strain, 285 genes were downregulated and 233 genes upregulated in the Δ*Rns1* mutant. ChIP-qPCR analysis showed that during cuticle penetration, RNS1 bound to the *BM2* motif in the promoters of two protease genes (*Pr1a* [MAA_05675] and *Pr1b* [MAA_08168]) that were regulated by RNS1 (see below) but not to the *BM1* motif in the promoter of MAA_10686 that was not regulated by RNS1 during cuticle penetration (Fig. S5D and E). Therefore, RNS1 only recognized the *BM2* motif during cuticle penetration, and we thus searched for this motif in the promoters of the differentially expressed genes profiled by the RNA-Seq analysis. Among the 285 downregulated genes in the Δ*Rns1* mutant, 262 had the *BM2* motif in their promoters, and this motif was also found in the promoters of 226 upregulated genes. Particularly, among the 262 downregulated *BM1* motif-containing genes, 28 encoded cuticle-degrading enzymes, including 15 for lipid utilization, 11 proteases, and 2 chitinases.

Cuticle-degrading enzymes are functionally redundant, and their expression has synergistic impacts (12); we thus investigated the extent to which RNS1 controlled overall activity of cuticle-degrading enzymes. Compared with that in the WT strain, the Δ*Rns1* mutant secreted significantly less Pr1 subtilisin protease activity (*P* < 0.05), though total extracellular protease activity was not altered in the mutant (see Fig. S6B and C). Likewise, the WT strain produced significantly more chitinases and lipases than the Δ*Rns1* mutant (Fig. S6D and E). We further quantified the cuticle degradation products following 12-h fungal growth in the cuticle medium. Although the WT strain had the same biomass as the Δ*Rns1* mutant, the mutant released significantly (*P* < 0.05) fewer amino acids from the cuticle (Fig. S6A, F, and G).

10.1128/mSystems.00499-21.6 FIG S6 RNS1 regulates cuticle-degrading enzymes during cuticle penetration. For quantification of free amino acids, peptides, and activities of cuticle degrading enzymes, mycelium grown in SDY medium for 36 h was transferred to cuticle medium using the locust cuticle as the sole carbon and nitrogen source. (A) Wet weight of mycelium after 12 h of growth in cuticle medium. Total extracellular protease (B), Pr1 protease (C), chitinase (D), and lipase (E) activity in the culture supernatant. Concentrations of peptides (F) and free amino acids (G) in culture supernatants. The control was cuticle medium that was not inoculated with the mycelium. All assays were repeated three times. Values with different lowercase letters are significantly different (*n* = 3, *P* < 0.05, Tukey’s test in one-way ANOVA). WT, WT strain; Δ*Rns1*, *Rns1* deletion mutant; C-Δ*Rns1*, complemented Δ*Rns1* strain. Download FIG S6, TIF file, 1.7 MB. Copyright © 2021 Meng et al. 2021 Meng et al. https://creativecommons.org/licenses/by/4.0/ This content is distributed under the terms of the Creative Commons Attribution 4.0 International license.

### The Fus3/RNS1 cascade regulates utilization of less-favored carbon and nitrogen sources.

As described above, RNS1 regulates the degradation of cuticular protein, chitin, and lipids, i.e., less-favored carbon and nitrogen sources. We thus postulated that it also regulated utilization of non-insect-derived less-favored carbon and nitrogen sources to support saprophytic growth. To test this postulation, we first compared mycelial dry weight of the WT with the that of the Δ*Rns1* mutant when they were grown for 48 h in the liquid medium containing less-favored carbon and nitrogen sources that were not derived from insects. As in other fungi, glucose is also a favored carbon source for Metarhizium fungi (21). It has not been documented which amino acids Metarhizium fungi prefer, and we assume that glutamine and ammonium are preferred by this group of fungi, because these two nutrients are reported to be favored nitrogen sources for many fungi (10). In this study, protein, lipids, and chitin are designated complex less-favored carbon or nitrogen sources, while some sugars such as raffinose are defined as less-favored carbon sources and amino acids such as arginine and proline are less-favored carbon and nitrogen sources. In the nutrient-rich medium SDY, the mycelial dry weight of the Δ*Rns1* mutant was the same as the WT strain (see Fig. S7A). In the medium with the non-insect-derived protein casein (named casein medium) or colloid chitin (chitin medium) as the sole carbon and nitrogen sources, the dry weight of the WT mycelium was significantly higher than that of the Δ*Rns1* mutant, but no significant difference was found between the two strains when proline, arginine, glutamine, or *N*-acetylglucosamine (the chitin monomer) was used as the sole carbon and nitrogen source (Fig. S7B and D). In the medium using the non-insect-derived lipid pentadecane (lipid medium) as the sole carbon source (ammonium was the nitrogen source), the mycelial dry weight of the Δ*Rns1* mutant was significantly lower than that of the WT strain (Fig. S7C), and no difference was found between these two strains when glucose, trehalose, or raffinose was used as the sole carbon source (Fig. S7C). In all media, no significant difference in mycelial dry weight was found between the WT strain and the complemented C-Δ*Rns1* strain (Fig. S7). On the nutrient-rich medium potato dextrose agar (PDA), the conidial yield of the Δ*Rns1* mutant was significantly lower than that of the WT strain and the complemented C-Δ*Rns1* strain. On the lipid, chitin, or casein medium, the WT strain still produced more conidia than the Δ*Rns1* mutant (Fig. S7E).

10.1128/mSystems.00499-21.7 FIG S7 Mycelial dry weight and conidial yield in different media. (A) Nutrient-rich medium SDY. Conidia (10^8^) were grown in 100 ml of the medium for 36 h. (B) Media using free amino acids or casein as the sole carbon and nitrogen source. (C) Media using sugars or the lipid pentadecane as the carbon source and ammonium as the nitrogen source. (D) Media using colloid chitin or *N*-acetylglucosamine (chitin monomer) as the sole carbon and nitrogen source. In panels B to D, the mycelium was first grown in SDY medium, which (0.2 g [wet weight]) was then inoculated into the specified media. After 48 h of growth at 26°C, the mycelial dry weight was measured. Values with different lowercase letters are significantly different (*P* < 0.05, Kruskal-Wallis test). (E) Conidial yield on PDA, lipid, casein, or chitin medium. WT, wild-type strain; Δ*Rns1*, *Rns1* deletion mutant; C-Δ*Rns1*, complemented Δ*Rns1* strain. Download FIG S7, TIF file, 2.8 MB. Copyright © 2021 Meng et al. 2021 Meng et al. https://creativecommons.org/licenses/by/4.0/ This content is distributed under the terms of the Creative Commons Attribution 4.0 International license.

**FIG 5:**
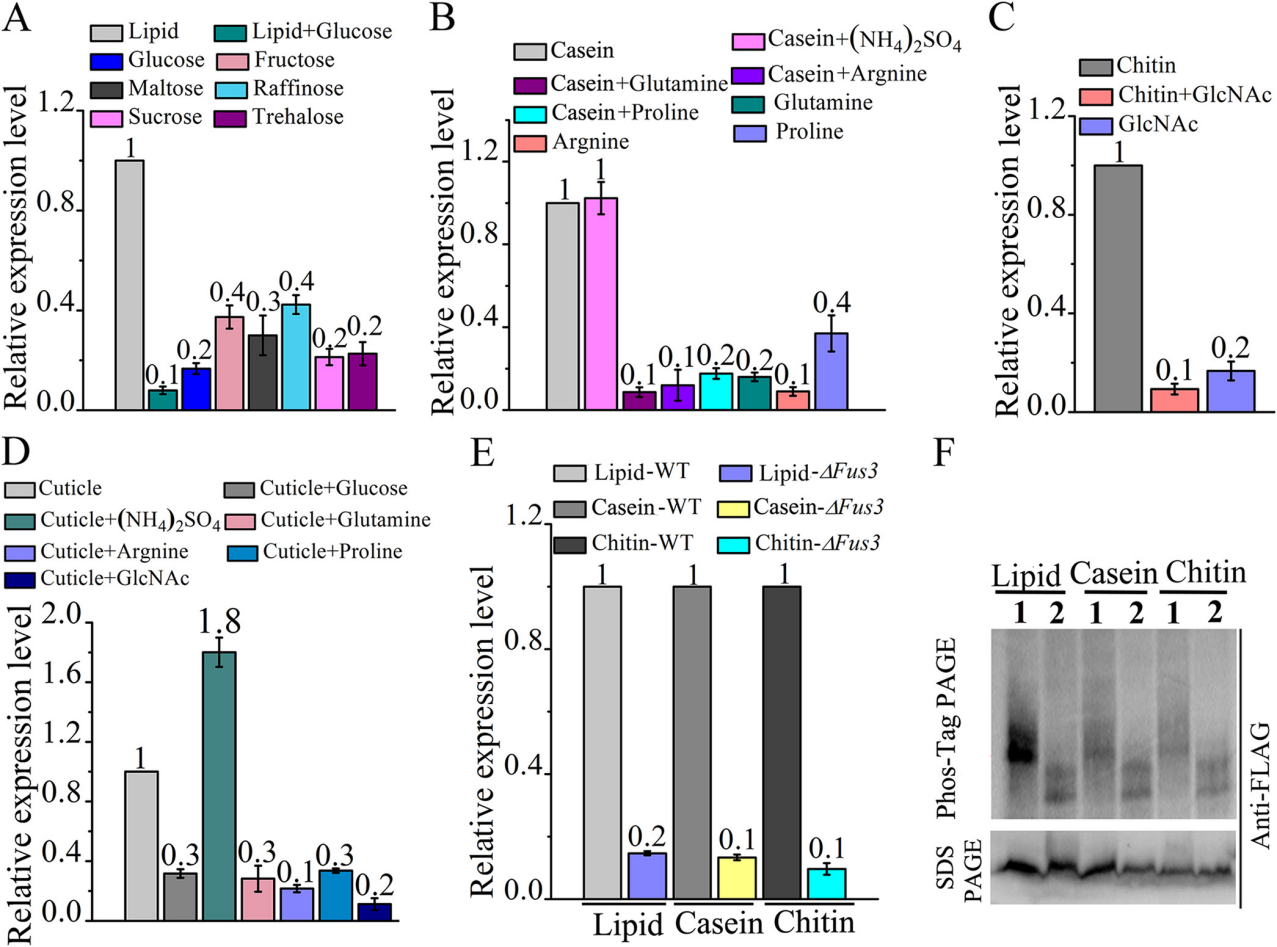
Fus3 regulates RNS1 during utilization of the non-insect-derived less-favored nutrients chitin, casein, and lipid (pentadecane is used as a representative of lipids). qRT-PCR analysis was conducted to analyze gene expression. (A) *Rns1* expression in the media with favored (glucose) and less-favored (lipid, fructose, maltose, trehalose, raffinose, and sucrose) carbon sources. The nitrogen source was (NH_4_)_2_SO_4_. (B) *Rns1* expression in the media with favored [glutamine, (NH_4_)_2_SO_4_] and less-favored (casein, proline, or arginine) carbon and nitrogen sources. (C) *Rns1* expression in the media containing chitin or *N*-acetylglucosamine (GlcNAc; the chitin monomer) as the sole carbon and nitrogen source. (D) *Rns1* expression in te cuticle medium with or without glucose, glutamine, proline, arginine, (NH_4_)_2_SO_4_, or *N*-acetylglucosamine. (E) *Rns1* expression in the WT strain and the Δ*Fus3* mutant when they were grown in lipid, chitin, or casein medium. (F) Phos-tag analysis of RNS1 phosphorylation in lipid, chitin, or casein medium. 1, *WT-RNS1-FLAG* strain; 2, Δ*Fus3-RNS1-FLAG* strain.

We then compared *Rns1* expression between the media with different carbon and nitrogen sources. The expression level of *Rns1* in the SDY medium was lower than in the lipid, casein, and chitin media, but no difference was found between the SDY medium and the media without carbon or nitrogen sources, i.e., under carbon or nitrogen starvation (see Fig. S8A). *Rns1* was more highly expressed in the lipid medium than in the medium using glucose, trehalose, maltose, sucrose, fructose, or raffinose as the sole carbon source, and no difference was found between these six sugar-containing media (Fig. 5A). Glucose suppressed *Rns1* expression in the lipid medium (Fig. 5A). *Rns1* was 5-fold more highly expressed in the casein medium than in the media with a free amino acid (glutamine, proline, or arginine) as the sole carbon and nitrogen source, and no difference was found between these amino acid-containing media. A free amino acid significantly reduced *Rns1* expression in the casein medium, but the inorganic favored nitrogen source ammonium had no impact on *Rns1* expression in the casein medium (Fig. 5B). In the chitin medium, the expression level of *Rns1* was 5-fold higher than that with *N*-acetylglucosamine as the sole carbon and nitrogen source, and *N*-acetylglucosamine inhibited *Rns1* expression in the chitin medium (Fig. 5C). Glucose, glutamine, arginine, proline, and *N*-acetylglucosamine all repressed *Rns1* expression in the cuticle medium (Fig. 5D), and again, ammonium had no impact on *Rns1* expression in the cuticle medium (Fig. 5D).

10.1128/mSystems.00499-21.8 FIG S8 RNS1 upregulates its own expression in media with non-insect-derived complex less-favored carbon and nitrogen sources (casein, chitin or lipids [pentadecane is used as a representative]). (A) qRT-PCR analysis of *Rns1* expression in SDY, lipid, casein, and chitin media and in the media without carbon or nitrogen source, i.e., carbon and nitrogen starvation. Expression of the GFP transcript (qRT-PCR analysis) (B) and GFP protein (Western blotting) (C) by the *WT-PRns1-gfp* strain in the media with different carbon and nitrogen sources. When glucose, raffinose, or lipid was used as the sole carbon source, the nitrogen source was ammonium. In other media, the sole carbon and nitrogen source was shown as their name: glutamine, glutamine was the sole carbon and nitrogen source; GlcNAc, *N*-acetylglucosamine. Expression of the GFP transcript (top) and protein (bottom) in six different strains when they were grown in lipid medium (D), in casein medium (E), and in chitin medium (F). Detailed description of the strains is shown in Table 1. Download FIG S8, TIF file, 2.9 MB. Copyright © 2021 Meng et al. 2021 Meng et al. https://creativecommons.org/licenses/by/4.0/ This content is distributed under the terms of the Creative Commons Attribution 4.0 International license.

Consistent with results regarding *Rns1* transcription (Fig. 5), in the *WT-PRns1-gfp* strain, the expression levels of GFP transcript and protein in the casein, chitin, or lipid medium were higher than in the media containing glucose, raffinose, glutamine, arginine, proline, or *N*-acetylglucosamine (Fig. S8B and C).

Compared with that in the WT strain, the expression level of *Rns1* was lower in the Δ*Fus3* mutant in the casein, chitin, or lipid medium (Fig. 5E). Also, in these three media, Phos-tag assays showed that Fus3 phosphorylated the RNS1 protein (Fig. 5F).

Another assay also showed that the Fus3 regulated *Rns1* expression for utilizing protein, chitin, and lipids. When grown in the casein, chitin, or lipid medium, the expression levels of the GFP transcript and protein in the *WT-PRns1-gfp* strain were significantly higher than in the *WT-PRns1*^Δ^*^BM2^-gfp*, Δ*Rns1-PRns1-gfp*, Δ*Rns1-PRns1*^Δ^*^BM2^-gfp*, Δ*Fus3-PRns1-gfp*, and Δ*Fus3-Pns1*^Δ^*^BM2^-gfp* strains (Fig. S8D to F), but no differences were found between the latter five strains (Fig. S8D to F).

### RNS1 regulates genes for utilizing protein, chitin, and lipids.

**FIG 6:**
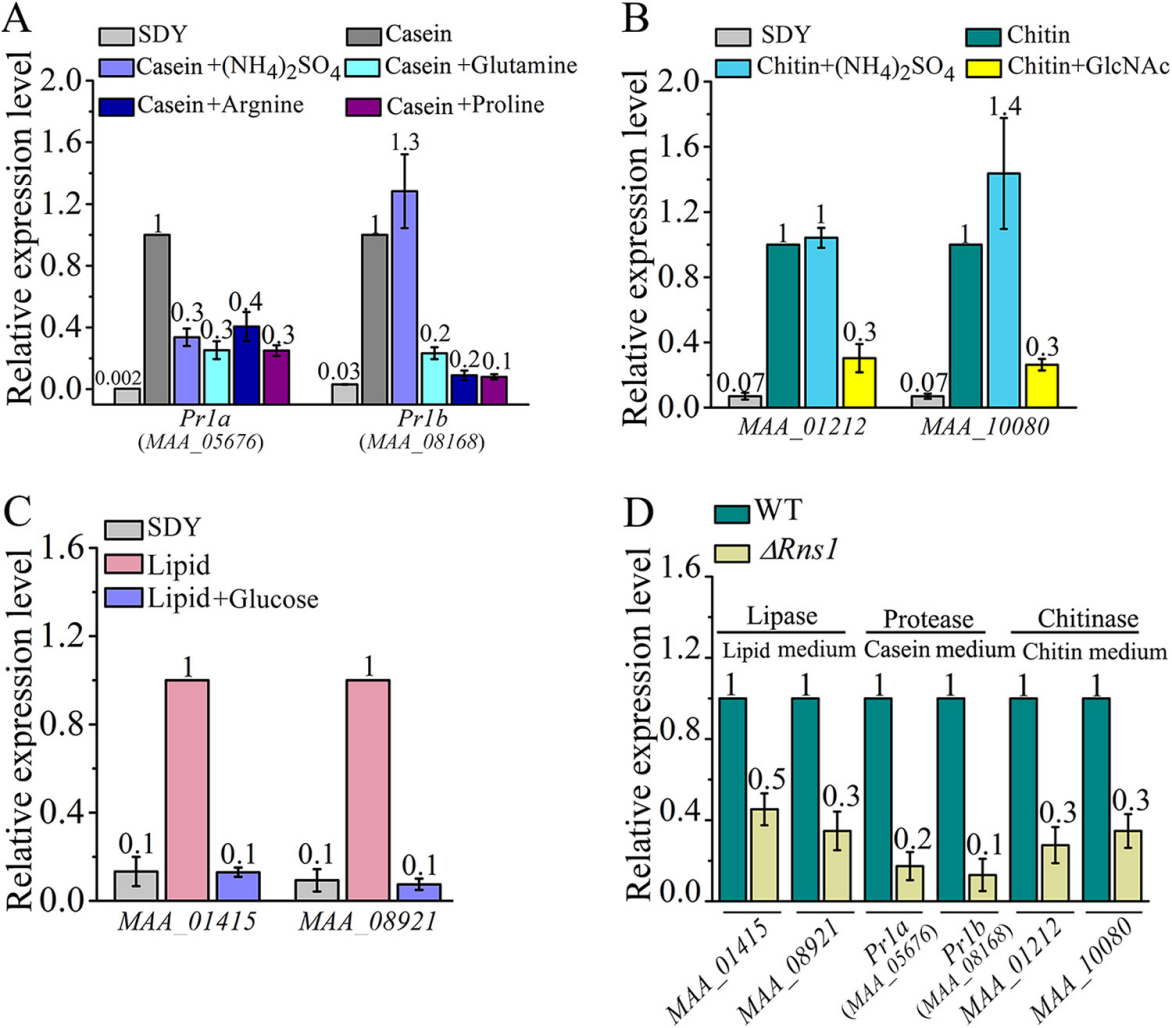
RNS1 regulates the expression of proteases, chitinases, and lipases for utilization of the protein casein, chitin, and lipids that are not derived from insects. qRT-PCR analysis was conducted to analyze gene expression. (A) Expression of two protease genes (*Pr1a* and *Pr1b*) in SDY (SDY) and casein medium with or without free amino acids and ammonium. (B) Expression of two chitinase genes (MAA_01212 and MAA_10080) in SDY and the chitin medium with or without *N*-acetylglucosamine and ammonium sulfate. (C) Expression of two lipase genes (MAA_01415 and MAA_08921) in SDY medium and the lipid medium with or without glucose. (D) Expression in the Δ*Rns1* deletion mutant of the protease genes in casein medium, chitinase genes in chitin medium, and lipase genes in lipid medium relative to that in the WT strain.

We further investigated whether RNS1 regulated the genes for utilizing casein, chitin, and lipids. In the *M. robertsii* genome, there are genes encoding 34 lipases, 29 chitinases, and 122 proteases (11). The *BM2* motif was identified in the promoters of 29 chitinase genes, 34 lipase genes, and 107 protease genes. Using two proteases (Pr1a and Pr1b), two lipases (MAA_08921 and MAA_01415), and two chitinases (MAA_01212 and MAA_10080) as their representatives, we assayed whether RNS1 regulated these *BM2* motif-containing genes. Compared with that during growth in the SDY medium, the expression of these representative protease, chitinase, and lipase genes was upregulated in the casein, chitin, and lipid media, respectively (Fig. 6A, B, and C). Compared with expression in the WT strain, these representative genes were all downregulated in the Δ*Rns1* mutant (Fig. 6D). Ammonium had no impact on *Pr1b* expression in the casein medium but suppressed *Pr1a* expression. An amino acid (glutamine, arginine, or proline) repressed the expression of *Pr1a* and *Pr1b* in the casein medium (Fig. 6A). In the chitin medium, ammonium had no impact on the expression of two chitinase genes, but *N*-acetylglucosamine inhibited their expression (Fig. 6B). Glucose inhibited the expression of the two lipase genes in the lipid medium (Fig. 6C).

### Relationship between RNS1 and other regulators of carbon and nitrogen metabolism.

We further investigated the relationship between RNS1 and other previously known regulators of carbon and nitrogen metabolism. We identified the regulator AREA (MAA_03820) in NMR, and CRR1 (MAA_06444) in CCR, and constructed their deletion mutants, Δ*AreA* and Δ*Crr1* (Fig. S2D and E). In the cuticle medium with or without glucose, no differences in *Rns1* expression were found between the WT strain and the Δ*Crr1* mutant (see Fig. S9A). Since RNS1 upregulated the lipase genes MAA_08921 and MAA_01415 in the lipid medium (Fig. 6C) and glucose suppressed their expression (Fig. S9C), these two lipase genes were used as indicators to assay whether RNS1 regulated CRR1-mediated glucose repression. qRT-PCR analysis showed that CRR1 mediated glucose repression of MAA_08921, but RNS1 had no impact on this repression (Fig. S9C). Both CRR1 and RNS1 had no impact on the glucose repression of MAA_01415 (Fig. S9C). In the lipid medium, the mycelial dry weight of the WT strain was significantly higher than that of the Δ*Crr1* mutant, which was in turn higher than that in the Δ*Rns1* mutant (Fig. S9D).

10.1128/mSystems.00499-21.9 FIG S9 The Fus3/RNS1 cascade is not directly related to other known regulators of carbon and nitrogen metabolism. (A) *Rns1* expression in the WT strain and the Δ*Crr1* mutant in cuticle medium with or without glucose. Unless otherwise indicated, gene expression presented in this figure was analyzed by qRT-PCR analysis. (B) *Rns1* expression in the Δ*AreA* and Δ*Snf1* mutants and the knockdown mutants of the gene *Tor* (*Tor^kd^*), *Tps1* (*Tps1^kd^*), and *G6PD* (*G6PD^kd^*) in cuticle medium. (C) Expression of two lipase genes in the WT strain and the Δ*Rns1* and Δ*Crr1* deletion mutants in lipid medium with or without glucose. (D) Mycelial dry weight of the WT strain and the Δ*Rns1*, Δ*Crr1* and Δ*AreA* mutants in lipid, casein, or chitin medium. Values with different lowercase letters are significantly different (*P* < 0.05, Kruskal-Wallis test). (E) NAD^+^ production when the WT and Δ*Rns1* strains were grown in SDY medium or insect cuticle medium. (F) RNA-Seq analysis of the expression of 10 *Nmr* genes in the WT and Δ*Rns1* strains in cuticle medium. The expression level of a gene in the #1 repeat of the WT strain is set to 1; the values represent the log_2_-transformed fold changes of differential gene expression in other treatments. #1, #2, and #3, three repeats. Expression of two protease genes in casein medium (G) and two chitinase genes in chitin medium (H) in the WT strain and the Δ*Rns1* and Δ*AreA* mutants. (I) Expression of five proline utilization genes in the WT strain and the Δ*Rns1* and Δ*AreA* mutants in the medium with proline as the sole carbon and nitrogen source. *PrnA*, MAA_08437 (GenBank accession number); *PrnB*, MAA_01021; *PrnC*, MAA_07805; *PrnD*, MAA_07804; *PrnX*, MAA_10225. (J) Expression in the WT strain and the Δ*Rns1* mutant of previously reported regulators (*AreA*, *Crr1*, *Snf1*, *Tps1*, *Tor*, and *G6PD*) in cuticle medium. Download FIG S9, TIF file, 2.9 MB. Copyright © 2021 Meng et al. 2021 Meng et al. https://creativecommons.org/licenses/by/4.0/ This content is distributed under the terms of the Creative Commons Attribution 4.0 International license.

In the cuticle medium, no difference in *Rns1* expression was found between the WT and the Δ*AreA* mutant (Fig. S9B). The protease genes (*Pr1a* and *Pr1b*) and the chitinases (MAA_01212 and MAA_10080) were used as indicators to test if RNS1 regulated AREA to control genes for utilizing alternative carbon and nitrogen sources. In the casein medium, no difference in *Pr1b* expression was found between the WT strain and the Δ*AreA* mutant, but both strains had higher expression levels than the Δ*Rns1* mutant (Fig. S9G). *Pr1a* was more highly expressed in the WT strain than in the Δ*AreA* and Δ*Rns1* mutants, and no difference was found between these two mutants (Fig. S9G). In the casein medium, the mycelial dry weight of the Δ*Rns1* mutant was the same as that of the Δ*AreA* mutant (Fig. S9D). In the chitin medium, the two chitinase genes were regulated by *Rns1* (Fig. S9H) but not by *AreA*; the mycelial dry weight of the Δ*AreA* mutant was the same as that of the Δ*Rns1* mutant but was lower than that of the WT strain (Fig. S9D). AREA positively regulated four proline utilization genes, but no difference in expression of these four genes was found between the WT strain and the Δ*Rns1* mutant (Fig. S9I). In the cuticle medium, the WT strain had the same NAD^+^ level as the Δ*Rns1* mutant (Fig. S9E), and RNA-Seq analysis showed that no difference in expression of the 10 *Nmr* genes was found between the WT and Δ*Rns1* strains (Fig. S9F).

In addition to AREA and CRR1, the Tps1 pathway (9), Snf1 (22), and a TOR kinase (23) have been reported to regulate fungal carbon and nitrogen metabolism. The genes encoding Snf1 (MAA_04401), the TOR kinase (MAA_02388), and two genes in the Tps1 pathway (*Tsp1* [MAA_04676], and *G6PD* [MAA_00144]) were also identified in *M. robertsii*. In the cuticle medium, the expression levels of *Tsp1*, *G6PD*, *Snf1*, and *Tor* in the WT strain were not different from the those in the Δ*Rns1* mutant (Fig. S9J). The deletion mutant of *Snf1* and the knockdown mutants of *Tor*, *Tps1*, and *G6PD* were constructed (Fig. S2F and G), and in the cuticle medium, no significant difference in *Rns1* expression was found between the WT strain and the mutants (Fig. S9B).

## DISCUSSION

The entomopathogenic fungus *M. robertsii* secretes numerous enzymes to degrade cuticular lipids, protein, and chitin and thereby obtains nutrients for hyphal growth and enters the hemocoel for further colonization. The Fus3-MAPK cascade regulates cuticle-degrading genes and is indispensable for cuticle penetration (14). In this study, we found that Fus3 activated the expression of the cuticle-degrading genes via the transcription factor RNS1. On the insect cuticle, Fus3 phosphorylates RNS1. The phosphorylated RNS1 migrates into the nucleus, binds to its own promoter, and self-upregulates its transcription, which then induces the expression of the cuticle-degrading genes. The deletion mutant of the *Fus3* gene was nonpathogenic (14), whereas deleting *Rns1* only resulted in partial loss of virulence. Therefore, RNS1 regulates a subset of pathogenicity genes controlled by the Fus3-MAPK cascade.

On an inductive milieu, single-celled conidia of *M. robertsii* need external nutrients for germination and following hyphal growth. The infection structure appressorium then forms on the tip of a multicellular hypha (15). Deleting *Rns1* suppressed the expression of cuticle-degrading genes and reduced the ability to exploit the cuticular lipids, protein, and chitin as nutrients and thereby slowed hyphal growth and delayed appressorial formation. In addition to the cuticle-degrading genes, RNA-Seq analysis showed that *Rns1* also regulated many other genes, but none of them were previously characterized genes related to appressorium formation. *Rns1* is also not involved in appressorial turgor pressure. Therefore, it remains to be resolved whether *Rns1* regulates appressorial formation itself. When *M. robertsii* reaches the hemocoel where free amino acids and sugars such as trehalose and glucose are available, these nutrients suppress *Rns1* expression, explaining why no significant difference in virulence was observed between the WT strain and the Δ*Rns1* mutant when inoculation was conducted by direct injection of conidia into the hemocoel. This is consistent with our previous findings that the Fus3-MAPK cascade is not involved in hemocoel colonization (14).

In addition to insect cuticular lipids, protein, and chitin, *Rns1* also regulates exploitation of non-insect-derived less-favored carbon and nitrogen sources as nutrients for saprophytic growth. RNS1 differs in two aspects from the transcription factor AREA. First, RNS1 is only involved in utilization of the complex less-favored carbon and nitrogen sources (proteins and chitin), whereas in addition to the complex carbon and nitrogen nutrients, AREA also regulates utilization of less-complex alternative carbon and nitrogen sources such as free amino acids. Second, the favored nitrogen sources affect AREA and RNS1 activity differently. Both ammonium and glutamine inhibit AREA activity (3). Glutamine also suppresses RNS1 activity, but ammonium’s impacts on RNS1 vary depending on its target genes. Ammonium had no impact on RNS1-mediated upregulation of *Pr1b* and two chitinase genes, whereas it suppressed the induction of *Pr1a* by RNS1. Nevertheless, both RNS1 and AREA activated *Pr1a* expression, and their mutants showed reduced mycelial growth in the casein medium, suggesting RNS1 and AREA have overlap in controlling the utilization of alternative nitrogen sources. However, RNS1 and AREA did not regulate each other’s transcription, and RNS1 also did not control two other components (NAD^+^ and *nmr* genes) in NMR. Therefore, the relationship between RNS1 and NMR remains to be resolved. Genetic and transcriptional analysis also failed to identify the relationship between RNS1 and other regulators of fungal carbon and nitrogen metabolism, including CRR1, Tps1, Snf1, and TOR kinase.

In conclusion, the Fus3-MAPK and the transcription factor RNS1 constitute a regulatory cascade that activates the utilization of the complex less-favored nitrogen and carbon sources by *M. robertsii*. This cascade enables this fungus to degrade cuticular protein, lipid, and chitin to obtain nutrients for hyphal growth and enter the hemocoel for further infection. The Fus3-MAPK cascade exists across the fungal kingdom, and RNS1 homologs are also widely found in ascomycete filamentous fungi, including saprophytes and pathogens with diverse hosts, suggesting that regulation of the utilization of alternative nitrogen and carbon sources by the Fus3/RNS1 cascade could be widespread.

## MATERIALS AND METHODS

### Gene deletion and gene knockdown.

*M. robertsii* ARSEF 2575 was obtained from the Agricultural Research Service Collection of Entomopathogenic Fungi (US Department of Agriculture). E. coli DH5α was used for plasmid construction. Agrobacterium tumefaciens AGL1 was used for fungal transformation as previously described (24).

Gene deletion and complementation of the deletion mutants were performed as previously described (25). Gene knockdown using the antisense RNA method was performed as described previously (12). Briefly, an ∼200-bp DNA fragment corresponding to part of the coding sequence of a target gene was inserted downstream of the constitutive promoter *Ptef* in the plasmid pPK2-TEF (15) to form pPK2-bar-GFP-RNAi, which was then transformed into *M. robertsii* mediated by A. tumefaciens. Gene knockdown was confirmed by qRT-PCR analysis. All primers used in this study are presented in Table S1 in the supplemental material.

10.1128/mSystems.00499-21.10 TABLE S1 Primers used in this study. Download Table S1, DOCX file, 0.03 MB. Copyright © 2021 Meng et al. 2021 Meng et al. https://creativecommons.org/licenses/by/4.0/ This content is distributed under the terms of the Creative Commons Attribution 4.0 International license.

### Pathogenicity assays.

Pathogenicity assays were conducted using last-instar G. mellonella larvae (RuiQing Bait Co., Shanghai, China). Inoculations were performed by topical application of conidia on the insect cuticle or by injection of conidia into the insect hemocoel (26). Mortality was recorded daily, and the LT_50_ values were determined using the SPSS statistical package (SSPS, Chicago, IL, USA). All bioassays were repeated three times with 40 insects per repeat.

Appressorial formation on the hydrophobic surface of a petri dish (Corning, NY, USA) was assayed as previously described (15). Turgor pressure of appressorium was measured as previously described (27).

### Assays of mycelial growth and conidial yield.

To assay the mycelial growth in SDY medium, 10^8^ conidia were inoculated into 100 ml of SDY and cultured at 26°C for 36 h with 160-rpm shaking. The mycelium was then collected by filtration and dried by lyophilization, and the mycelial dry weight was then measured. To measure mycelial growth in other media, the mycelium collected from the SDY medium (0.3 g [wet weight]) was inoculated into a medium. After 48 h of growth at 26°C, the mycelial dry weight was measured.

### Yeast two-hybrid assays.

The interaction between RNS1 and Fus3 was assayed using yeast two-hybrid assays (Clontech, Japan). The coding sequences of RNS1 and Fus3 were cloned by PCR and inserted into the plasmids pGADT7 and pGBKT7 to produce the plasmids pGADT7-RNS1 and pGBKT7-Fus3, respectively. The plasmid pGBKT7-Fus3 was transformed into Y2HGold cells, and pGADT7-RNS1 was transformed into Y187 cells. The resulting strain from mating was grown on medium (SD-His-Ade-Leu-Trp) supplemented with X-α-Gal (5-bromo-4-chloro-3-indolyl-α-d-galactopyranoside) and aureobasidin A (AbA; TaKaRa Bio). The autoactivation of Fus3 was tested by inoculating the strain containing the plasmid pGBKT7-Fus3 on the medium (SD-His-Ade-Trp) with X-α-Gal. Yeast two-hybrid and autoactivation assays were repeated three times.

### Co-IP analysis.

The master plasmid pPK2-sur-Ptef-FLAG or pPK2-bar-Ptef-HA was used for producing a protein fused with FLAG or HA, respectively (26). To assay the *in vivo* interaction between RNS1 and Fus3, an *RNS1-FLAG/Fus3-HA* strain expressing the fusion protein RNS1::FLAG and Fus3::HA was constructed. The coding sequence of RNS1 was inserted into pPK2-sur-Ptef-FLAG, and the resulting plasmid was then transformed into the WT strain to produce the *WT-RNS1-FLAG* strain. The coding sequencing of Fus3 was inserted into the plasmid pPK2-bar-Ptef-HA to produce pPK2-sur-Ptef-Fus3-HA, which was transformed into the *WT-RNS1-FLAG* strain to produce the *RNS1-FLAG/Fus3-HA* strain.

Extraction of total fungal proteins and Co-IP analysis were performed as described previously (26). Protein concentration was determined using the bicinchoninic acid (BCA) protein assay kit (Meilune, Dalian, China). The mouse anti-HA antibody was purchased from Abclonal (Hangzhou, China), and the rabbit anti-FLAG antibody was purchased from Sigma-Aldrich (MO, USA). Dynabeads protein G beads were purchased from Invitrogen (CA, USA). Detection of a target protein was conducted using Western blot analysis. All blots were imaged by a chemiluminescence detection system (Clarity Western ECL; Bio-Rad). All Co-IP assays were repeated three times.

### BiFC assay.

The coding sequences of YFP^N^ (Met-1 to Asp-174) and YFP^C^ (Gly-175 to Lys-239) were cloned by PCR as described previously (28), and the resulting PCR products were inserted into the BamH I/EcoRV sites of pPK2-Ptef-bar and pPK2-Ptef-sur to produce the master plasmids pPK2-bar-Ptef-YFP^N^ and pPK2-sur-Ptef-YFP^C^, respectively. The coding sequences of RNS1-DBD (Glu-61 to Pro-267) and Fus3 were inserted into the Bam I/Eco I sites of pPK2-bar-Ptef-YFP^N^ and pPK2-sur-Ptef-YFP^C^, respectively, to produce pPK2-bar-Ptef-YFP^N^-RNS1-DBD and pPK2-sur-Ptef-YFP^C^-Fus3. The resulting plasmids were transformed into the WT strain to produce the *RNS1-DBD-YFP^N^/Fus3-YFP^C^* strain. As a control, the plasmids pPK2-bar-Ptef-YFP^N^-RNS1-DBD and pPK2-sur-Ptef-YFP^C^ were transformed into the WT strain to produce the *RNS1-DBD-YFP^N^/YFP^C^* strain. YFP fluorescence was analyzed using a Leica TCS SP2 laser confocal scanning microscope (Germany).

### Phos-tag analysis.

The mutagenesis kit (NEB, UK) was used to construct the mutated RNS1 proteins RNS1^T215A^, RNS1^S226A^, and RNS1^T215A/S226A^ (described in Results).

The mycelium cultured in SDY medium was used for analysis of protein phosphorylation during saprophytic growth. Since it was not possible to obtain enough biomass to get sufficient protein for Phos-tag analysis by growing *M. robertsii* on the locust hindwings, we prepared the mycelium by growing the fungus in cuticle medium (basal salt medium containing 1% locust cuticle as sole carbon and nitrogen sources). To do this, the mycelium from the SDY medium was collected by filtration, washed with ample sterile water three times, and then grown in the locust cuticle medium for 1 h with 160-rpm shaking.

The Phos-tag analysis was conducted as described previously (29). The mycelium (∼0.7 g) was ground into fine powder in liquid nitrogen and then suspended in 3 ml of lysis buffer (26). After being incubated at 4°C for 6 to 8 h, the mixture was centrifuged, and the supernatant was used for Phos-tag analysis. An SDS-PAGE (sodium dodecyl sulfate-polyacrylamide gel electrophoresis) gel (8%) containing 25 μM Phos binding reagent acrylamide (APExBIO, USA) and 100 μM MnCl_2_ was used. After electrophoresis, proteins in the gel were blotted to a polyvinylidene difluoride (PVDF) membrane (Bio-Rad, USA) at 100 V for 6 h, which was then subjected to Western blotting using the anti-FLAG antibody (Huabio, China).

### ChIP-Seq and ChIP-qPCR analyses.

ChIP-Seq analysis was conducted by the company Igenebook (Wuhan, China). The anti-FLAG antibody was used for immunoprecipitation, and DNA fragments enriched by ChIP were sequenced on an Illumina HiSeq 2000. After removing sequencing adaptors and low-quality bases, the clean reads were mapped to the *M. robertsii* genome using the software BWA (version 0.7.15-r1140) (30). The peak caller MACS (version, 2.1.1.20160309; *q* value < 0.05) was used to localize the potential binding sites of RNS1 (31).

ChIP-qPCR analysis was conducted as previously described (26). ChIP-enriched DNA was used as a template for quantitative PCR analysis using Thunderbird SYBR qPCR mix without ROX (Toyobo, Japan). All ChIP-qPCR analyses were repeated three times.

### Expression and preparation of RNS1-DBD in E. coli.

To express RNS1-DBD in E. coli, its coding sequence was inserted into the BamHI/EcoRI sites of the plasmid pET-SUMO (Invitrogen, USA), and the resulting plasmid was then transformed into E. coli BL-21. The expression of the recombinant protein was induced by isopropyl β-d-1-thiogalactopyranoside at 18°C for 16 h (Novagen, Madison, WI, USA). The fusion protein SUMO::RNS1-DBD was partially purified with HisPur Ni-nitrilotriacetic acid (NTA) resin (Thermo Fisher Scientific, MA, USA), and the SUMO tag was then removed with laboratory-prepared SUMO protease ULP1. The recombinant RNS1-DBD protein was further purified to homogeneity with HisPur Ni-NTA resin.

### EMSA.

To prepare a biotin-labeled double-stranded DNA probe, one of the DNA strands was biotin-labeled and annealed to its complementary strand (unlabeled) according to the manufacturer’s instructions (TsingKe Biological Technology, Hangzhou, China). To prepare an unlabeled WT DNA probe, two regular DNA strands were commercially synthesized and annealed to form the probe. A mutated DNA probe was prepared exactly as the WT DNA probe with one nucleotide in the RNS1 binding motif replaced with each of the other three. EMSAs were conducted using the LightShift Chemiluminescent EMSA kit (Thermo Fischer Scientific, MA, USA). In the competition assays, an unlabeled DNA probe was added in a 200-fold excess.

### RNA-Seq and qRT-PCR analyses.

Total RNA was extracted with TRIzol reagent (Life Technologies, USA). RNA-Seq analysis was conducted by Personal Gene Technology (Nanjing, China). Paired-end sequencing was performed on an Illumina HiSeq 2000 sequencing platform. Clean reads were mapped to the *M. robertsii* genome using software HISAT2. Reads that aligned uniquely to the reference sequence were used for gene expression quantification using the fragments per kilobases per million fragments (FPKM) method. Differential expression analysis was performed with DESeq software with an adjusted *P* value of 0.05 (Benjamini-Hochberg method).

For qRT-PCR analysis, cDNAs were synthesized with total RNAs using ReverTra Ace qPCR RT master mix (Toyobo, Osaka, Japan). qRT-PCR analysis was conducted using Thunderbird SYBR qPCR mix without ROX (Toyobo). The reference genes *Gpd* and *tef* were used as described previously (32). The relative expression level of a gene was calculated using the comparitive threshold cycle (2^−ΔΔ^*^CT^*) method (33). All qRT-PCR experiments were repeated three times.

### Assay of subcellular localization of RNS1-DBD.

To assay the impact of phosphorylation of RNS1 by Fus3 on its cellular localization, GFP-tagged proteins RNS1-DBD::GFP, RNS1^T215A^-DBD::GFP, and RNS1^S226A^-DBD::GFP were constructed. To do this, the coding sequences of RNS1-DBD, RNS1^T215A^-DBD, and RNS1^S226A^-DBD were individually inserted into the BamHI/EcoRI sites of the plasmid pPK2-Ptef-GFP-N (26), and the resulting plasmids were transformed into the WT strain to produce *WT-RNS1-DBD-GFP*, *WT-RNS1^T215A^-DBD-GFP*, and *WT-RNS1^S226A^-DBD-GFP* strains and into the Δ*Fus3* mutant to form Δ*Fus3-RNS1-DBD-GFP*, Δ*Fus3-RNS1^T215A^-DBD-GFP*, and Δ*Fus3-RNS1^S226A^-DBD-GFP* strains. The expression and integrity of the fusion proteins were analyzed with Western blot analysis using anti-GFP antibody (Huabio, Hangzhou, China). The subcellular localization of a fusion protein was determined by following the GFP signal. The nuclear was visualized by DAPI (4′,6-diamidino-2-phenylindole) staining.

### Quantification of amino acids and peptides and assays of the activities of cuticle-degrading enzymes.

Quantification of amino acids and peptides released during enzymolysis of the insect cuticle was conducted as previously described (12). Briefly, the mycelium (1 g [wet weight]), collected from the SDY medium, was inoculated into 100 ml of the cuticle medium, followed by 12 h of incubation at 26°C with 160-rpm shaking. Free amino acids in the supernatant were quantified with an amino acid quantification kit (Solarbio, Shanghai), and peptides were quantified using the Bradford assay kit (Bio-Rad, USA). The activity of the total extracellular proteases was assayed using the Azocasein kit (Sigma, USA). One unit of proteolytic activity was defined as an increase of 0.01 absorbance at 440 nm after 1 h of incubation at 28°C. Pr1 subtilisin protease activity was assayed using the specific substrate Suc-(Ala)_2_-Pro-Phe-*p*-nitroanilide (NA) (Sigma, USA). One unit of activity was defined as the amount of enzyme that released 1 μmol of NA per minute at 28°C. The activity of chitinase was assayed using the chitinase assay test kit (Solarbio Life Sciences, Beijing, China). One unit of chitinase activity was defined as the amount of enzyme that released 1 μg of *N*-acetylglucosamine per minute at 37°C. Lipase activity assay was conducted as described previously (34). 4-Nitrophenyl palmitate was used as the substrate to measure the activity of lipases. One unit of enzyme activity was defined as the amount of enzyme to produce 1 μmol of *p*-nitrosophenol per min at 37°C.

### Extraction of promoter sequences from *M. robertsii* genome.

The promoter sequence of a gene was extracted based on the reference genome of *M. robertsii* using an in-house Perl script. The promoter of a gene was determined as the 2-kb DNA fragment upstream of its open reading frame (ORF) start site or as the region, if shorter than 2 kb, between its ORF and the ORF of its adjacent gene.

### Data availability.

RNA-Seq data from the Δ*Rns1* mutant (accession number PRJNA720174 [SRR14339752, SRR14339744, and SRR14657472]) and the WT strain (PRJNA637940 [SRR11946811, SRR11947115, SRR11947195]) were deposited in the GenBank database.

## Supplementary Material

Reviewer comments
