# Supplementary figures and images for: A Novel Nitrogen and Carbon Metabolism Regulatory Cascade Is Implicated in Entomopathogenicity of the Fungus Metarhizium robertsii
Source: mSystems. 2021 Jun 22;6(3):e00499-21. doi: 10.1128/mSystems.00499-21 (PMC8269237; doi:10.1128/mSystems.00499-21)

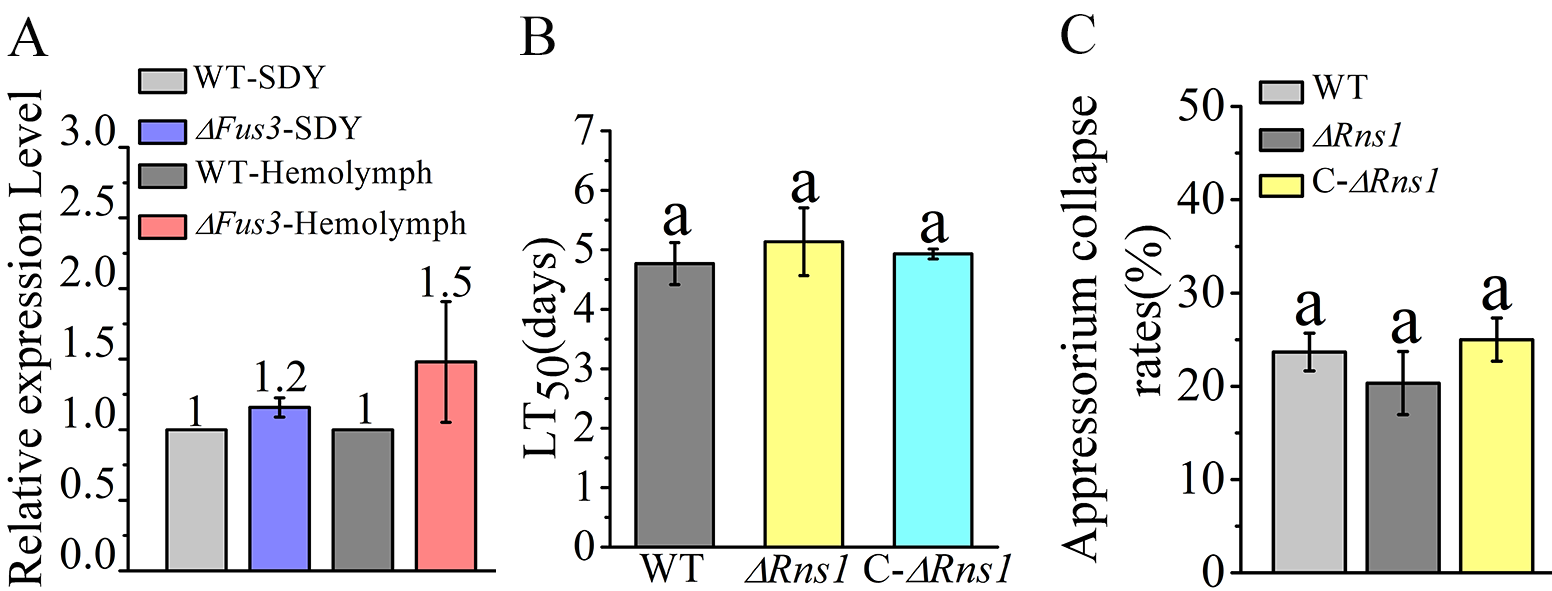

Supplement: FIG S1 [file msystems.00499-21-sf001.tif]

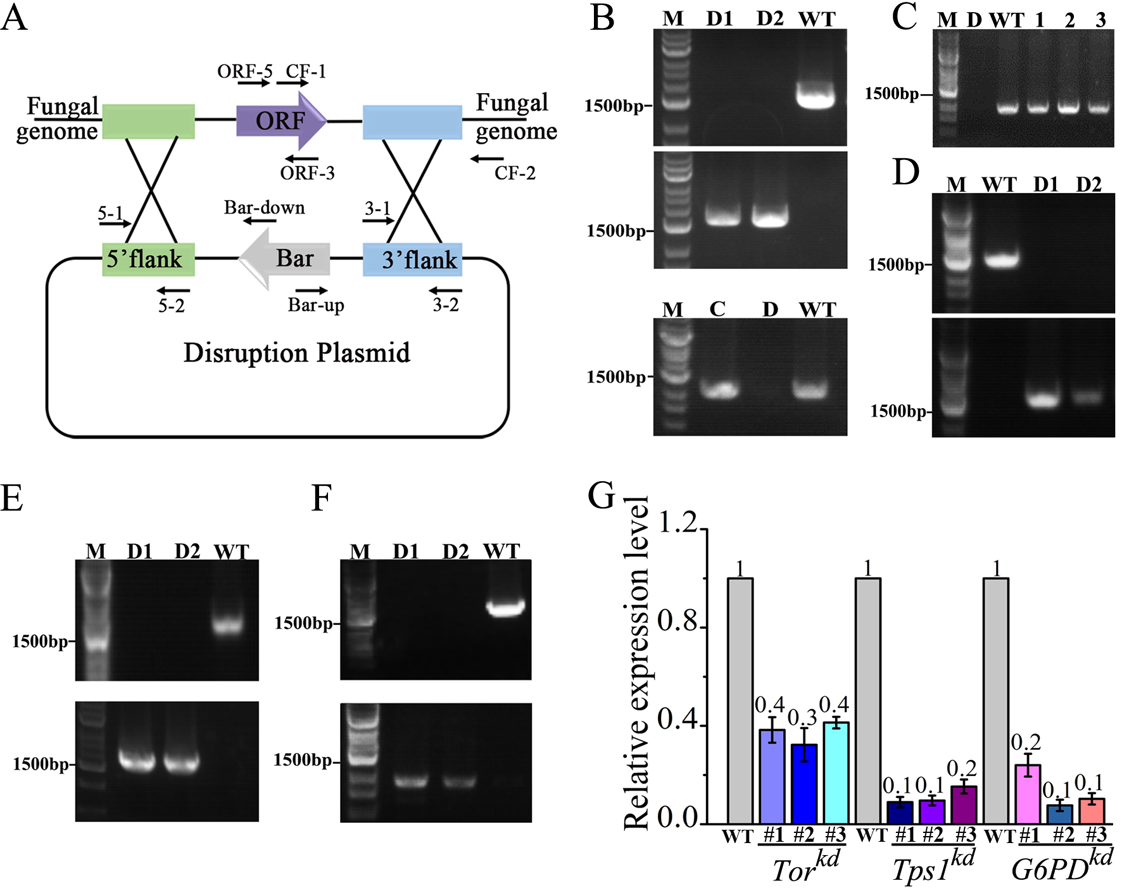

Supplement: FIG S2 [file msystems.00499-21-sf002.tif]

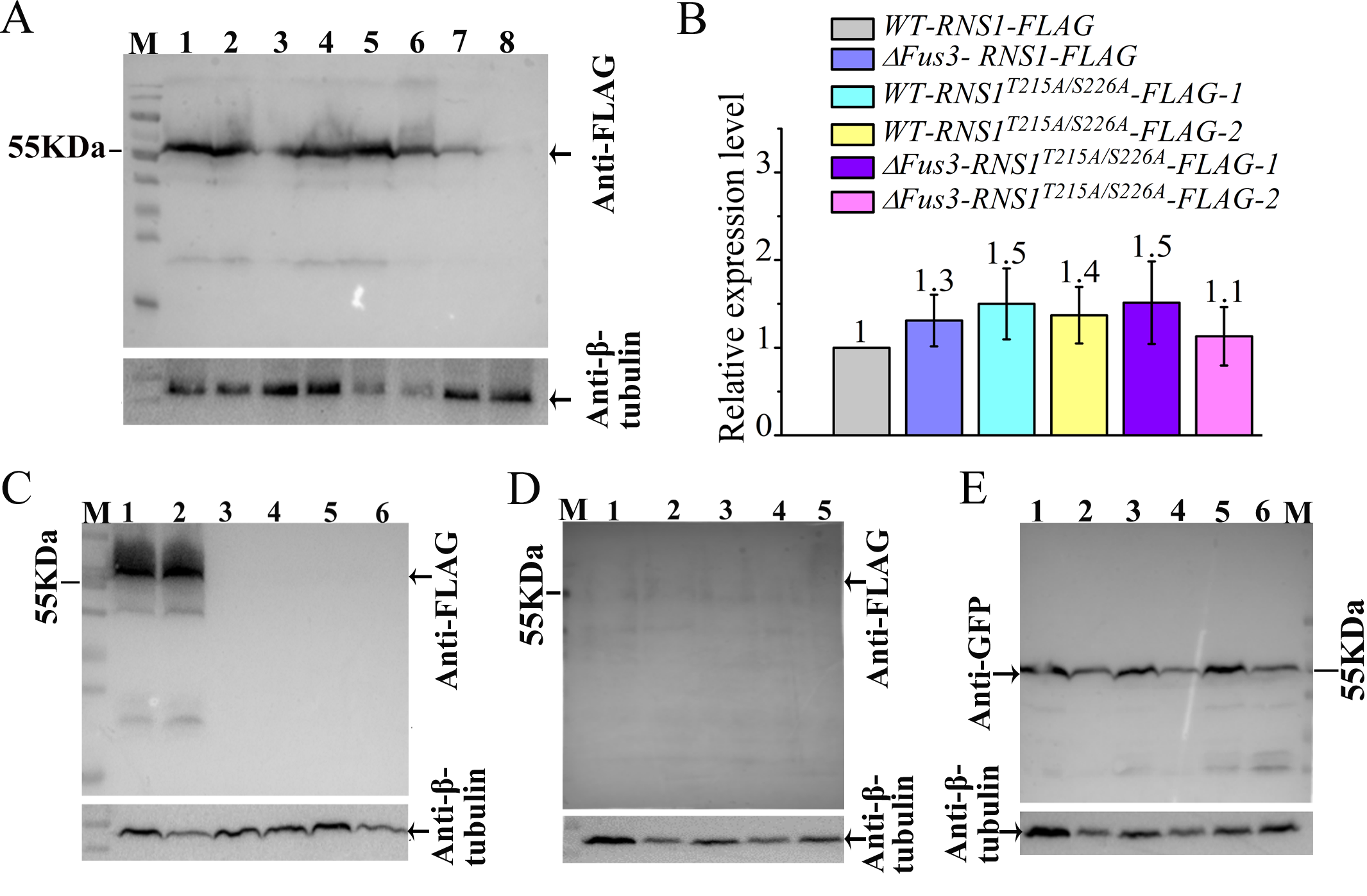

Supplement: FIG S3 [file msystems.00499-21-sf003.tif]

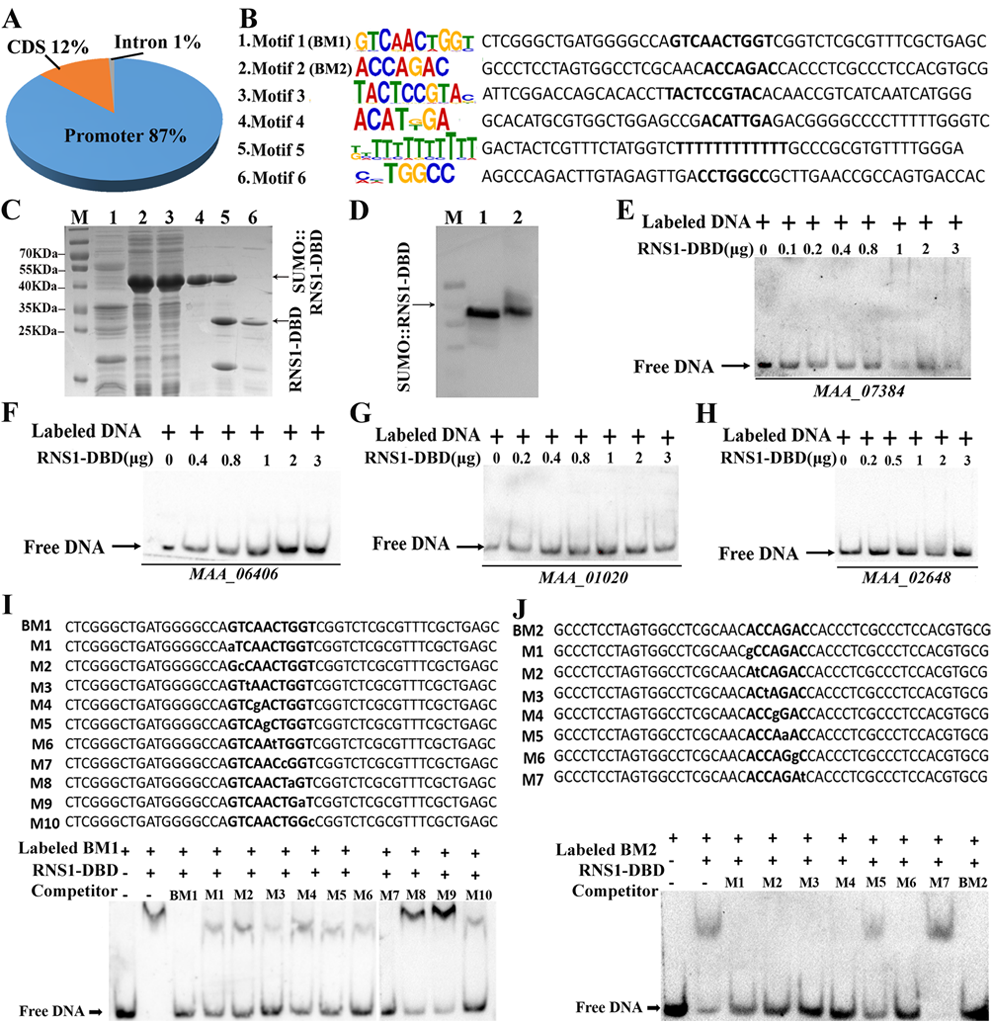

Supplement: FIG S4 [file msystems.00499-21-sf004.tif]

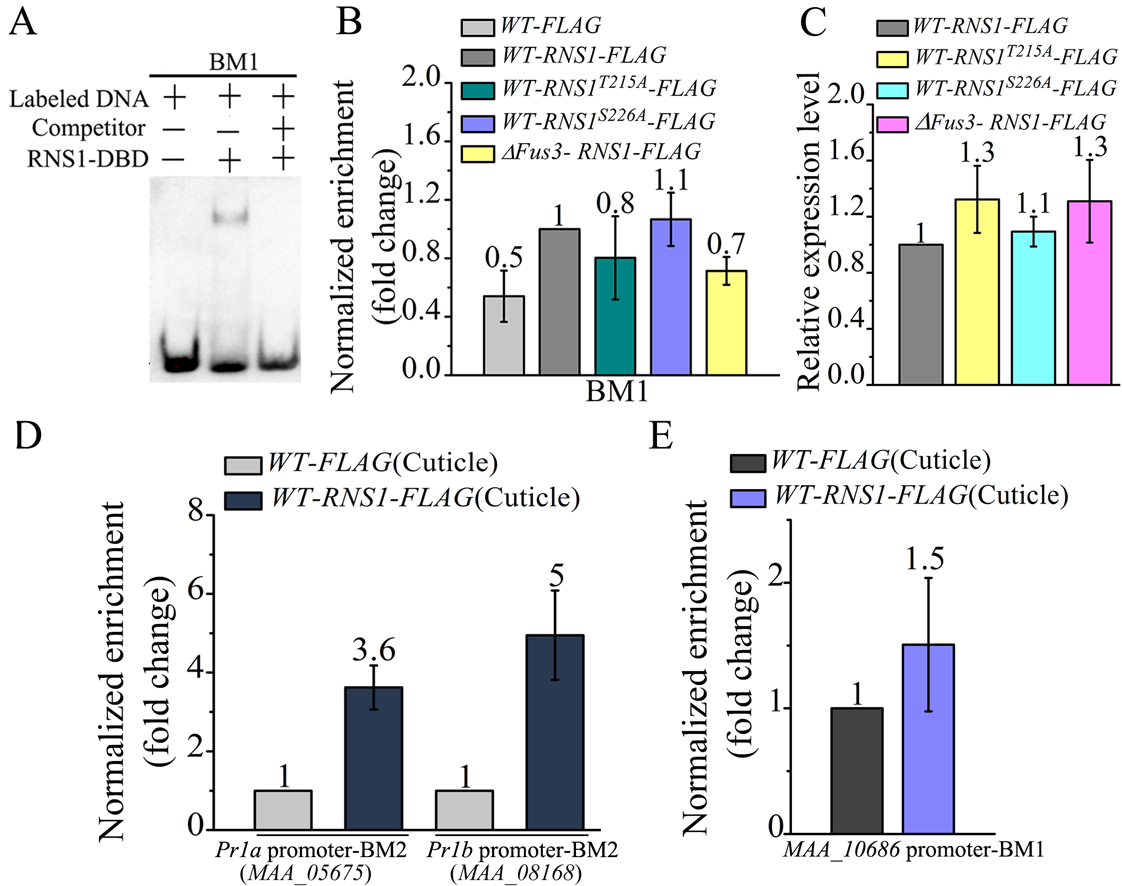

Supplement: FIG S5 [file msystems.00499-21-sf005.tif]

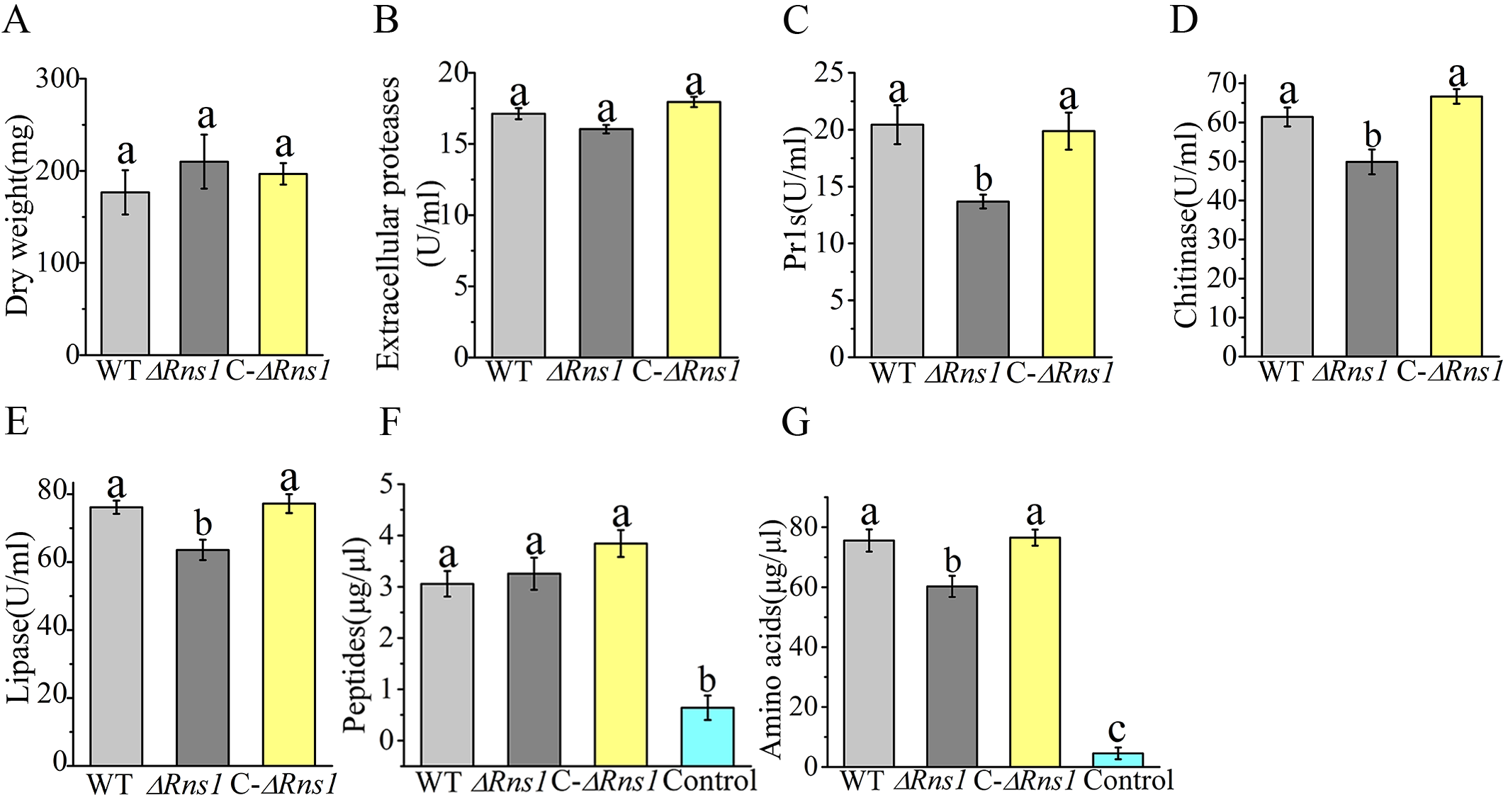

Supplement: FIG S6 [file msystems.00499-21-sf006.tif]

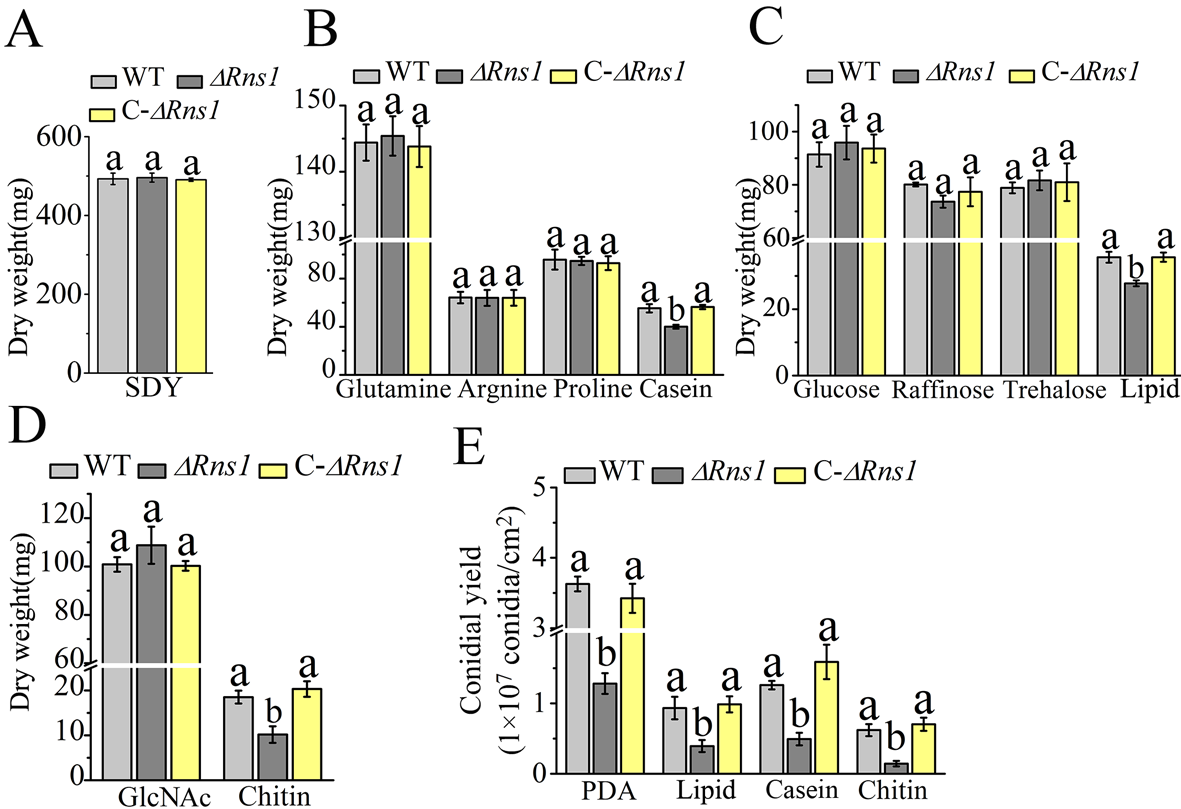

Supplement: FIG S7 [file msystems.00499-21-sf007.tif]

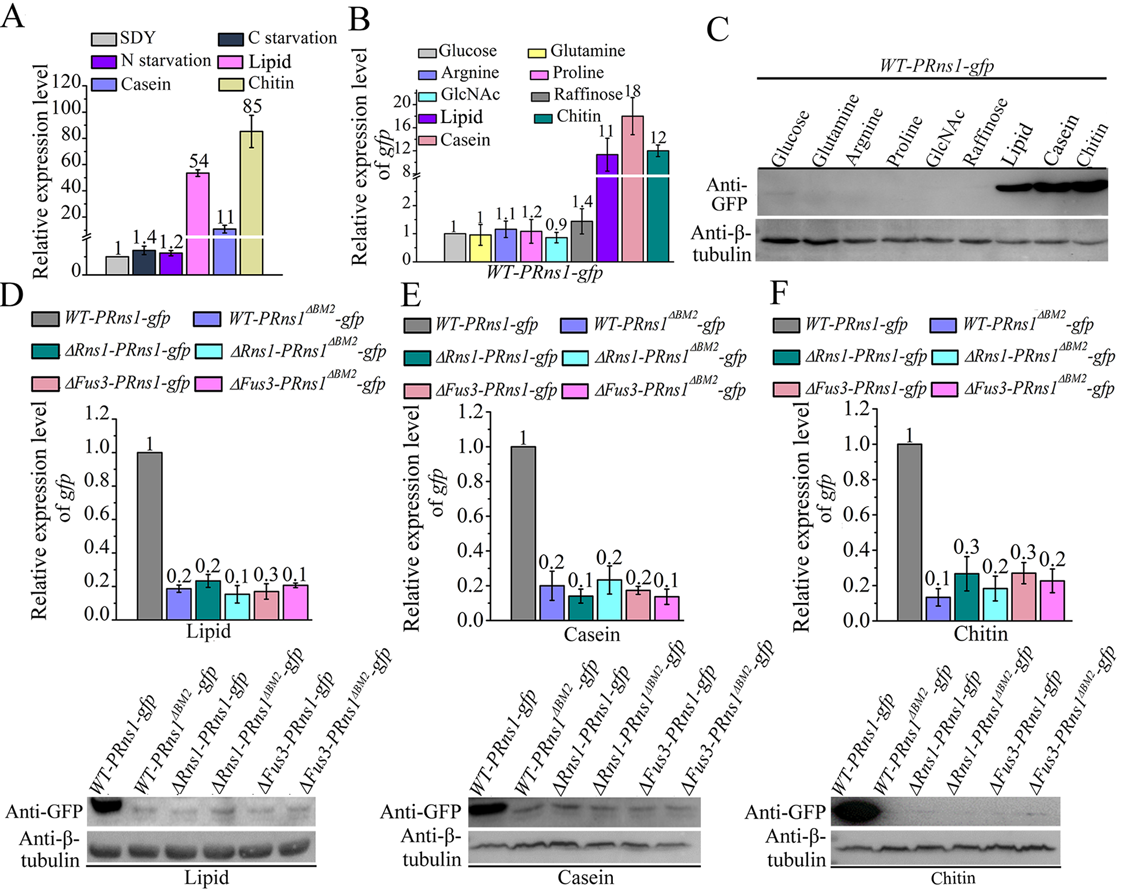

Supplement: FIG S8 [file msystems.00499-21-sf008.tif]

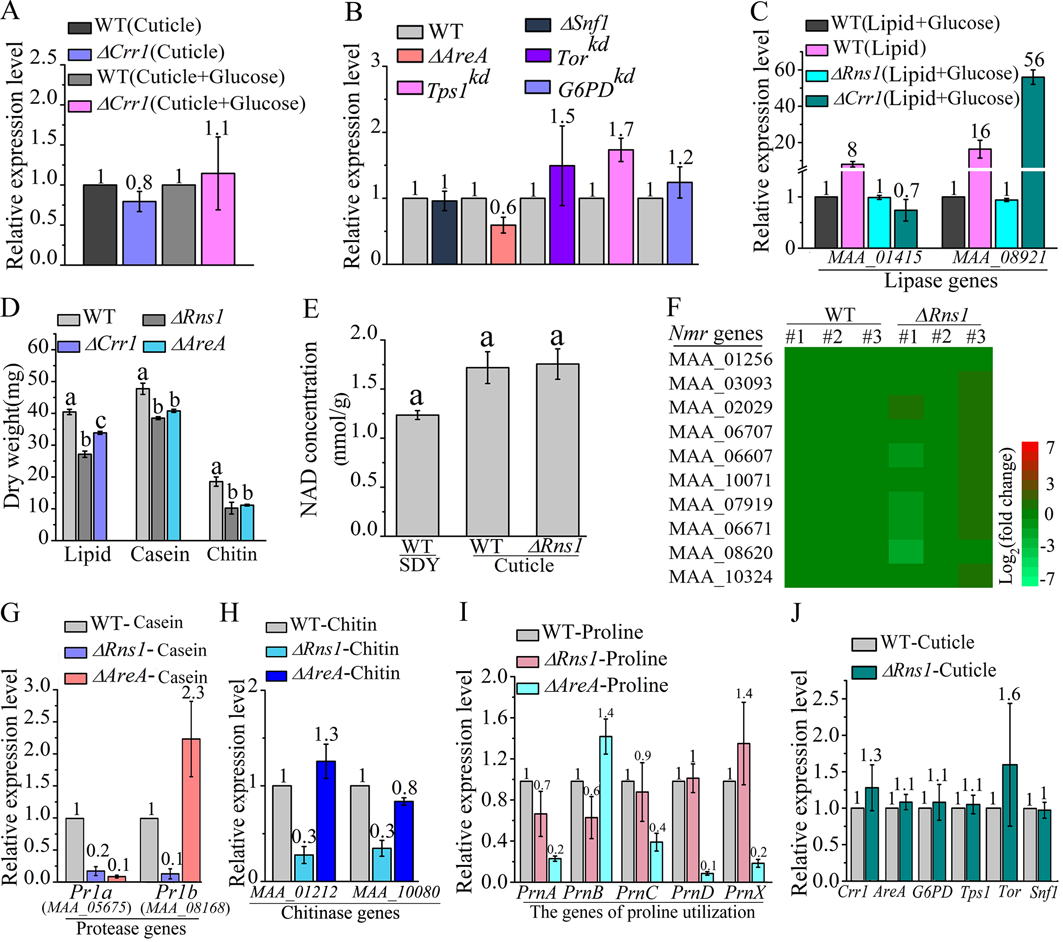

Supplement: FIG S9 [file msystems.00499-21-sf009.tif]
