# Supplementary material for: A Novel Nitrogen and Carbon Metabolism Regulatory Cascade Is Implicated in Entomopathogenicity of the Fungus Metarhizium robertsii
Source: mSystems. 2021 Jun 22;6(3):e00499-21. doi: 10.1128/mSystems.00499-21 (PMC8269237; doi:10.1128/mSystems.00499-21)
Supplement: TABLE S1 [file msystems.00499-21-st001.docx]

**Table S1**. Primers used in this study

| Primer name | Sequence | Usage |
| --- | --- | --- |
| DRNS1-5-1 | GGGGACAGCTTTCTTGTACAAAGTGGAAACGCTCAACTGTCCAAC | Disruption of *Rns1* |
| DRNS1-5-2 | GGGGACTGCTTTTTTGTACAAACTTGTAAGAGACCAGCTCTGTG |  |
| DRNS1-3-1 | GGGGACAACTTTGTATAGAAAAGTTGTTTGCTAGTCATGCTTGCC |  |
| DRNS1-3-2 | GGGGACAACTTTGTATAATAAAGTTGTACACCTTACTAGGTACC | Confirmation of the disruption of *Rns1* |
| DRNS1-CF-1 | CACAGGTACGCCTCCAG |  |
| DRNS1-CF-2 | CTAATACTCTTGCCTAG |  |
| DCRR1-5-1 | GGGGACAGCTTTCTTGTACAAAGTGGAACAGACTTCAGATGCCTG | Disruption of *Crr1* |
| DCRR1-5-2 | GGGGACTGCTTTTTTGTACAAACTTGTCTGCACTGCACTGGATC |  |
| DCRR1-3-1 | GGGGACAACTTTGTATAGAAAAGTTGTTTTGCGATGGTTACTATC |  |
| DCRR1-3-2 | GGGGACAACTTTGTATAATAAAGTTGTCTACTACTATCGTTCTC |  |
| DCRR1-CF-1 | AGGTCGCTGTTCAGGAC | Confirmation of the disruption of *Crr1* |
| DCRR1-CF-2 | ACGTCTCGCAACACTAG |  |
| DAreA-5-1 | GGTCTAGAGTTGGCGCTCATGCAAA | Disruption of *AreA* |
| DAreA-5-2 | GGGAATTCGAGACCAGGGACCAGTC |  |
| DAreA-3-1 | GGACTAGTTGAAAACCGATGTTATC |  |
| DAreA-3-2 | GGGATATCTCTGGCAAGTCCGTACT |  |
| DAreA-CF-1 | CGAGTCGCCCGTCTTCT | Confirmation of the disruption of *AreA* |
| DAreA-CF-2 | ATATTACATGTTTTTGTT |  |
| DSnf1-5-1 | GGTCTAGATCACGTTGAACAATCGC | Disruption of *Snf1* |
| DSnf1-5-2 | GGGAATTCCGTTCAGATTATCGTCC |  |
| DSnf1-3-1 | GGACTAGTGATGAATGAAGGCTAAC |  |
| DSnf1-3-2 | GGGATATCGGACGATAATCTGAACG |  |
| DSnf1-CF-1 | GCTTAATTCCCAGCCGCCTC | Confirmation of the disruption of *Snf1* |
| DSnf1-CF-2 | AAGTACACAACCGACGTGTG |  |
| Tps1-kd-5 | gggatatctacgaatacattgccacg | Knock down *Tps1* |
| Tps1-kd-3 | ggggatcctgggctattctgcgtaggg |  |
| TOR kinase-kd-5 | ggggatccccagaagctacacca | Knock down *Tor* |
| TOR-kinase-kd-3 | Gggatatccgccggcattcaatctta |  |
| G6PD-kd-5 | ggggatcccaacttgtttggtggtgc | Knock down *G6PD* |
| G6PD-kd-3 | gggatatccagactgtggttactgag |  |
| C-RNS1-5 | ATGCCTAAACATCATCG | Cloning genomic clone *gRns1* for complementation |
| C-RNS1-3 | CTACCGGCGGGCGGAAC |  |
| Bar-up | CGCCTGGACGACTAAACC | Confirmation of gene disruption |
| Bar-down | TCAGCCTGCCGGTACCGC |  |
| pET28a-RNS1-DBD-5 | ggggatccATGGAGCCATACGACGCCTA | Cloning RNS1-DBD for expression in *E.coli.* |
| pET28a-RNS1-DBD-3 | gggaattcTTAgtcgccgccgccgccga |  |
| RNS1-DBD-5 | ggggatccATGGAGCCATACGACGCCTA | Cloning RNS1-DBD for expression in *M. robertsii* . |
| RNS1-DBD-3 | gggaTATcTTAgtcgccgccgccgccga |  |
| RNS1-5 | ggGGTACCATGCCTAAACATCATCG | Cloning the CDS of RNS1 for expression in *M. robertsii* . |
| RNS1-3 | ggGTCGACCTACCGGCGGGCGGAAC |  |
| Fus3-5 | GGGAATTCATGTCCCGTTCGAACCC | Cloning the CDS of Fus3 for expression in *M. robertsii* . |
| Fus3-3 | GGGATATCTTACCTCATAATCTCCT |  |
| pGBKT7-Fus3-5 | GGGAATTCATGTCCCGTTCGAACCC | Cloning the CDS of Fus3 for Yeast two-hybrid |
| pGBKT7-Fus3-3 | GGGGATCCTTACCTCATAATCTCCT |  |
| pGADT7-RNS1-5 | ggGAATTCATGCCTAAACATCATCG | Cloning the CDS of RNS1 for Yeast two-hybrid |
| pGADT7-RNS1-3 | ggGGGATCCCGGGCGGAAC |  |
| YFP^N^-5 | ggagatctATGGTGAGCAAGGGCGA | Cloning the N terminus of YFP for BiFC assays |
| YFP^N^-3 | gggatatctctagagaattcggatcc GTTCTTCTGCTTAGGCC |  |
| YFP^C^-5 | ggagatctATGGGCATCAAGGTGAA | Cloning the C terminus of YFP for BiFC assays |
| YFP^C^-3 | gggatatctctagagaattcggatcc CTTGTACAGCTCGTCCA |  |
| T215A-mutant-5 | CATCGGCCTGgctCCTGTTGATG | Substituting the Thr-215 to alanine in RNS1 protein |
| T215A-mutant-3 | CCGGAAATGCCGCTGTCG |  |
| S226A-mutant-5 | ATCCTACAGCgctCCTGAGACGAGC | Substituting the Ser-226 to alanine in RNS1 protein |
| S226A-mutant-3 | GCGTCTAGTTCATCAACAG |  |
| BM2-mutant-5 | aaaggcctctcttggtttctc | Substituting all 7 nt to A in the *BM2* motif of *PRns1.* |
| BM2-mutant-3 | ttttatccgaaatcccgacttg |  |
| RNS1-ChIP-BM1-5 | CTACACGGCTCCATGAAAGA | ChIP-qPCR analysis for RNS1 |
| RNS1-ChIP-BM1-3 | CGACGATGGGCGAGCACGTT |  |
| RNS1-ChIP-BM2-5 | GCGTCAGTTTCATGAGGAAA |  |
| RNS1-ChIP -BM2-3 | GCCATCTGCGTCGCCAAGTT |  |
| MAA_10686-ChIP -5 | AGAGGCATCAGTTGATCGAG | ChIP-qPCR analysis for MAA_10686 |
| MAA_10686-ChIP-3 | GCATGACATGGGCCTTGGGG |  |
| MAA_05782-ChIP-5 | CCGTCGACGTCTGGACGCCT | ChIP-qPCR analysis for MAA_05782 |
| MAA_05782-ChIP-3 | AATGACGAATGGGACCGGGA |  |
| MAA_05675-ChIP-BM2-1-5 | GATGCTCGGGCAGATGGGCG | ChIP-qPCR analysis for Pr1a |
| MAA_05675-ChIP -BM2-1-3 | AGACTGTTTGTTGTCGGTAA |  |
| MAA_05675-ChIP-BM2-2-5 | CAGCTTCCAGTTCTGGAAGG |  |
| MAA_05675-ChIP -BM2-2-3 | ACCGAGGTTGATTTGATGGA |  |
| MAA_08168-ChIP-BM2-1-5 | CTTCCAATTCAGCTAATAAT | ChIP-qPCR analysis for Pr1b |
| MAA_08168-ChIP -BM2-1-3 | CATAAAGGATGCATCGTCA |  |
| MAA_08168-ChIP-BM2-2-5 | GCTGCAACTATTTCCCTCCT |  |
| MAA_08168-ChIP -BM2-2-3 | CACTCGTTAGTTCCATCGCA |  |
| RNS1-RT-5 | CCGACATCAAGCAGGACATG | qRT-PCR analysis for RNS1 |
| RNS1-RT-3 | CTTGTTCTTGATGCCTCGGG |  |
| GFP-RT-5 | ATCATGGCCGACAAGCAGAA | qRT-PCR analysis for GFP |
| GFP-RT-3 | TCTCGTTGGGGTCTTTGCTC |  |
| MAA_05197-RT-5 | AACAAAGGGTAGGACGCTCG | qRT-PCR analysis for MAA_05197 |
| MAA_05197-RT-3 | CGTCCCGGCTGACTAGAATC |  |
| MAA_08096-RT-5 | AGCCAACTCGACAATCGTGT | qRT-PCR analysis for MAA_08096 |
| MAA_08096-RT-3 | TGCTCGTCAACATTGTCCGA |  |
| MAA_06244-RT-5 | gagtgcacacaacaggagga | qRT-PCR analysis for MAA_06244 |
| MAA_06244-RT-3 | cttggccatcttcgaccctt |  |
| MAA_01415-RT-5 | cactcgtcagaccagccttt | qRT-PCR analysis for MAA_01415 |
| MAA_01415-RT-3 | ttccagagcccttcaacgtc |  |
| MAA_08921-RT-5 | agaacaacatgggctaccgg | qRT-PCR analysis for MAA_08921 |
| MAA_08921-RT-3 | tcaagcagagacgacaccac |  |
| MAA_05675-RT-5 | TCAGGCTGAGAGCATCATTG | qRT-PCR analysis for Pr1a |
| MAA_05675-RT-3 | CATCACAGCGTCCTTCTCAA |  |
| MAA_08168-RT-5 | GCCGGGTGTGAGTATCTTGT | qRT-PCR analysis for Pr1b |
| MAA_08168-RT-3 | GATGGCACATTCGTCAACAC |  |
| MAA_01212-RT-5 | TTGGCTTTTCTGAGCCACTT | qRT-PCR analysis for MAA_01212 |
| MAA_01212-RT-3 | TGCTTTGGCAGTTCTTGATG |  |
| MAA_10080-RT-5 | tcttcggtcagtgttcacgg | qRT-PCR analysis for MAA_10080 |
| MAA_10080-RT-3 | gactgcccacaaaacagctg |  |
| CRR1-RT-5 | ggtggagcgtgagactttga | qRT-PCR analysis for CRR1 |
| CRR1-RT-3 | caattggggtgtgatccgga |  |
| AreA-RT-5 | caggcggcacaatttcaaca | qRT-PCR analysis for AreA |
| AreA-RT-3 | ggtggagacggttgattggt |  |
| G6PD-RT-5 | cgaaacgagctggttatgcg | qRT-PCR analysis for G6PD |
| G6PD-RT-3 | ggtagtggagcaggggagta |  |
| Tps1-RT-5 | cgagctcgccgatagacatt | qRT-PCR analysis for Tps1 |
| Tps1-RT-3 | ttcttggtaggctgcccatg |  |
| Snf1-RT-5 | ccgaatgctcctgttcaggt | qRT-PCR analysis for Snf1 |
| Snf1-RT-3 | gctcttggccctgattgact |  |
| TOR kinase-RT-5 | tgtcttgctggagtgagctg | qRT-PCR analysis for TOR kinase |
| TOR kinase-RT-3 | cgccgctactttgatcttgc |  |
| Tef-RT-5 | CTGGTACAAGGGTTGGGAGA | Reference gene for qRT-PCR analysis |
| Tef-RT-3 | TACACATCCTGGAGGGGAAG |  |
| Act-RT-5 | TCCTGACGGTCAGGTCATC | Reference gene for qRT-PCR analysis |
| Act-RT-3 | CACCAGACATGACGATGTTG |  |
